# Reducing *Plasmodium falciparum* Malaria Transmission in Africa: A Model-Based Evaluation of Intervention Strategies

**DOI:** 10.1371/journal.pmed.1000324

**Published:** 2010-08-10

**Authors:** Jamie T. Griffin, T. Deirdre Hollingsworth, Lucy C. Okell, Thomas S. Churcher, Michael White, Wes Hinsley, Teun Bousema, Chris J. Drakeley, Neil M. Ferguson, María-Gloria Basáñez, Azra C. Ghani

**Affiliations:** 1 MRC Centre for Outbreak Analysis & Modelling, Department of Infectious Disease Epidemiology, Imperial College London, London, England; 2 Department of Infectious Diseases, London School of Hygiene & Tropical Medicine, London, England; St. George's Hospital Medical School, United Kingdom

## Abstract

**Background:**

Over the past decade malaria intervention coverage has been scaled up across Africa. However, it remains unclear what overall reduction in transmission is achievable using currently available tools.

**Methods and Findings:**

We developed an individual-based simulation model for *Plasmodium falciparum* transmission in an African context incorporating the three major vector species (*Anopheles gambiae s.s.*, *An. arabiensis*, and *An. funestus*) with parameters obtained by fitting to parasite prevalence data from 34 transmission settings across Africa. We incorporated the effect of the switch to artemisinin-combination therapy (ACT) and increasing coverage of long-lasting insecticide treated nets (LLINs) from the year 2000 onwards. We then explored the impact on transmission of continued roll-out of LLINs, additional rounds of indoor residual spraying (IRS), mass screening and treatment (MSAT), and a future RTS,S/AS01 vaccine in six representative settings with varying transmission intensity (as summarized by the annual entomological inoculation rate, EIR: 1 setting with low, 3 with moderate, and 2 with high EIRs), vector–species combinations, and patterns of seasonality. In all settings we considered a realistic target of 80% coverage of interventions. In the low-transmission setting (EIR∼3 ibppy [infectious bites per person per year]), LLINs have the potential to reduce malaria transmission to low levels (<1% parasite prevalence in all age-groups) provided usage levels are high and sustained. In two of the moderate-transmission settings (EIR∼43 and 81 ibppy), additional rounds of IRS with DDT coupled with MSAT could drive parasite prevalence below a 1% threshold. However, in the third (EIR = 46) with *An. arabiensis* prevailing, these interventions are insufficient to reach this threshold. In both high-transmission settings (EIR∼586 and 675 ibppy), either unrealistically high coverage levels (>90%) or novel tools and/or substantial social improvements will be required, although considerable reductions in prevalence can be achieved with existing tools and realistic coverage levels.

**Conclusions:**

Interventions using current tools can result in major reductions in *P. falciparum* malaria transmission and the associated disease burden in Africa. Reduction to the 1% parasite prevalence threshold is possible in low- to moderate-transmission settings when vectors are primarily endophilic (indoor-resting), provided a comprehensive and sustained intervention program is achieved through roll-out of interventions. In high-transmission settings and those in which vectors are mainly exophilic (outdoor-resting), additional new tools that target exophagic (outdoor-biting), exophilic, and partly zoophagic mosquitoes will be required.

*Please see later in the article for the Editors' Summary*

## Editors' Summary

**Background:**

Half the world's population is at risk of malaria, and every year nearly one million people—mainly children living in sub-Saharan Africa—die from this mosquito-borne parasitic disease. Most malarial deaths are caused by *Plasmodium falciparum*, which is transmitted to people by mainly night-biting *Anopheles* mosquitoes. When infected mosquitoes feed on people, they inject sporozoites, a parasitic form that replicates inside human liver cells. After a few days, the liver cells release “merozoites,” which invade red blood cells where they replicate rapidly before bursting out and infecting more red blood cells. This increase in the parasitic burden causes malaria's characteristic fever. Infected red blood cells also release “gametocytes,” which infect mosquitoes when they take a blood meal. In the mosquito, the gametocytes multiply and develop into sporozoites, thus completing the parasite's life cycle. Malaria can be prevented by spraying the insides of houses (where most *Anopheles* species feed and rest) with insecticides (indoor residual spraying, IRS) and by sleeping under bed nets that have been treated with long-lasting insecticides (long-lasting insecticide nets, LLINs). Mass screening and treatment (MSAT) with effective antimalarial drugs can also reduce malaria transmission.

**Why Was This Study Done?:**

Early attempts to eradicate malaria (reduce its global incidence to zero) in the 1950s reduced the incidence of malaria to zero in some countries (malaria elimination) and greatly reduced malarial illnesses and deaths in others (malaria control). However, this eradication program was aborted in the 1970s in part because of emerging drug and insecticide resistance. Recently, the advent of artemisinin-based combination therapies and new insecticides and the prospect of a malaria vaccine have renewed interest in controlling, eliminating, and ultimately eradicating malaria. Consequently, in September 2008, the Roll Back Malaria Partnership launched the Global Malaria Action Plan, which aims to reduce malaria deaths to near zero by 2015. But are the currently available tools for reducing malaria transmission sufficient to control and eliminate malaria in Africa, the continent where most malaria deaths occur? In this study, the researchers use a new mathematical model of *P. falciparum* transmission to investigate this question.

**What Did the Researchers Do and Find?:**

The researchers' *P. falciparum* transmission model consists of “compartments” through which individuals pass as they become infected with parasites, develop immunity, become infectious to mosquitoes, and so on. The researchers used published data about parasite prevalence (the proportion of the population infected with parasites) and about relevant aspects of mosquito, parasite, and human biology, to estimate the chances of an individual moving between compartments. Finally, they used the model to explore the impact over 25 years of increased coverage of LLINs, IRS, and MSAT, and of a future vaccine on malaria transmission in six representative African settings. In a low-transmission setting, 80% coverage with LLINs reduced the parasite prevalence to below 1% in all age groups. In two moderate-transmission settings, LLIN scale-up alone failed to reach this target but the addition of IRS and MSAT drove the parasite prevalence below 1%. However, this combination of interventions did not control malaria in a moderate-transmission setting in which a mosquito species that bites and rests outside houses contributes to malaria transmission. Finally, in two high-transmission settings, parasite prevalence could be driven below 1% only by setting unrealistic coverage targets for existing interventions.

**What Do These Findings Mean?:**

This new mathematical model greatly simplifies the complex dynamics of malaria transmission and includes several assumptions about which there is considerable uncertainty. The findings of this study are not, therefore, firm predictions of the future of malaria control in specific settings. Nevertheless, they suggest that it should be possible to make large reductions in malaria transmission and the associated disease burden in Africa over the next 25 years using currently available tools. Specifically, in regions where transmission is low or moderate and mosquitoes mainly feed indoors, it should be possible to reduce parasite prevalence to less than 1% provided a sustained intervention program is achieved. Importantly, however, these findings suggest that in regions where malaria transmission is high or where mosquitoes rest and bite outside houses, new approaches will be needed to control and eliminate malaria.

**Additional Information:**

Please access these Web sites via the online version of this summary at http://dx.doi.org/10.1371/journal.pmed.1000324.

- Information is available from the World Health Organization on malaria (in several languages); the 2009 World Malaria Report provides details of the current global malaria situation
- The US Centers for Disease Control and Prevention provide information on malaria (in English and Spanish)
- Information is available from the Roll Back Malaria Partnership on its approach to the global control of malaria, including the Global Malaria Action Plan and a fact sheet on malaria in Africa
- MedlinePlus provides links to additional information on malaria (in English and Spanish)

## Introduction

Over the past five years, dramatic declines in malaria disease caused by *Plasmodium falciparum* have been reported across a range of settings within sub-Saharan Africa. These declines are associated with increased distribution of long-lasting insecticide-treated nets (LLINs) and with the switch from a failing drug regimen to artemisinin-based combination therapies (ACT) as first-line therapy [1]–[4]. Whilst this pattern of reducing disease is encouraging, there remain many countries within Africa that continue to have a high burden of disease and hence malaria remains a leading cause of mortality in children under five years of age [5]. Thus control of the disease, and ultimately elimination of the parasite in this continent, remain major public health goals.

Eradication of malaria was attempted in the 1950s under the auspices of the World Health Organization-led Global Malaria Eradication Program (GMEP) [6]. Notably, Africa was not formally included in this program despite clear evidence of the large disease burden within the continent at that time. However, elimination campaigns were subsequently undertaken on a smaller scale within Africa, most prominently in two areas of moderate to high transmission in Nigeria (the Garki project [7],[8]) and on the Kenyan/Tanzanian border (the Pare-Taveta project [9]), but also periodically in areas of lower transmission including the Kenyan highlands [10] and the island of Madagascar [11]. These campaigns included frequent insecticide spraying of houses to reduce the vector populations and rounds of mass treatment to reduce the human infectious reservoir. Whilst substantial declines in infection and disease were observed in all of these campaigns, the control measures were not sufficient to eliminate the parasite on a short time scale, and failure to sustain control programs inevitably led to rebound of infection and disease in later years. This under-performance was perceived as a lack of success by past eradication attempts, which may in part be attributed to over-optimism about what could have been achieved with the tools then available [12].

Two years ago, following a renewed commitment to malaria control from donor organizations, the focus shifted again to malaria eradication as an ultimate goal. Previously, many countries had already intensified their own malaria control programs with much success in reducing both the burden of disease and ongoing transmission [1]–[4],[13],[14]. However, Africa poses the biggest challenge to a global eradication initiative, given the heterogeneous yet ubiquitous nature of *P. falciparum* transmission across much of the continent. Levels of transmission in Africa range from absent or low in many urban areas, through epidemic outbreaks in the highlands, to highly seasonal or perennial transmission in rural areas [15],[16]. This variable transmission pattern is further complicated by local variation in the major *Anopheles* vector populations that sustain transmission (principally *An. gambiae s.l.* and *An. funestus*, although approximately 70 relevant species have been identified worldwide [17]). Of the 47 countries within sub-Saharan Africa, the majority are currently classified by WHO/Roll-Back Malaria as being in the control stage and thus need to scale up interventions to sustain control and reduce the burden of disease via a reduction in transmission [18]. On the northern borders of the continent, transmission is already low, with Egypt and Algeria in the elimination phase and Morocco and Mauritius having interrupted local transmission. Similarly, in the southernmost countries, a sustained move towards local control and potentially elimination in border areas has been agreed upon via cooperation with neighbouring countries (the “elimination eight”) [19]. On the island of Zanzibar, a highly successful control program has reduced transmission to very low levels. However, a recent assessment of the feasibility of moving to elimination concluded that, whilst it is technically feasible to reduce local transmission to zero in this setting, the resources, both financial and operational, required to sustain elimination in the face of repeated reintroduction from mainland Africa make this a difficult prospect [20].

Compared to the past campaigns in the 1950s, additional tools are now available which, combined with sustained policy commitment, may make local elimination achievable in some settings and can aid control of disease by dramatically reducing malaria prevalence in countries with high rates of ongoing transmission. These include new LLINs, which have increased killing effects on the vectors compared to traditional nets and are more durable [21]–[25], and ACTs, which, through their gametocytocidal effect, can impact transmission from humans to vectors [26],[27]. In addition, a pre-erythrocytic malaria vaccine, RTS,S, has shown promising results in Phase II trials [28]–[34] and could soon contribute to elimination programs. National control agencies have varying levels of resources but can rarely implement all major control interventions at a given time. Understanding how to choose policy that is appropriate to the local setting is therefore key to effective control. Whilst the efficacies of most interventions have been individually evaluated in the field, the impact of different combinations of these is not clear. Field trials will be important to inform control policies but will be able to test only a few of the combinations of interventions in a limited number of settings.

Mathematical models provide a tool with which to explore the expected impact of different interventions against malaria, both individually and in combination, on a range of program endpoints [26],[35]–[40]. Whilst simple models can provide important general insights, the heterogeneity in transmission intensity [41]–[43], the variability in vector species composition and associated bionomics [17],[35], and the seasonality in vector populations [44] are all important factors that affect the transmission potential of a site and the likely impact of intervention packages. We therefore constructed an individual-based simulation model which captures these key factors while remaining sufficiently mathematically tractable to enable the baseline model parameters to be rigorously fitted to data within a Bayesian framework. The model includes the suite of current tools most often employed by (or likely to be employed by) National Malaria Control Programs—namely, LLINs, IRS, ACTs in case treatment and in mass treatment campaigns, and a vaccine with characteristics similar to the RTS,S/AS01 vaccine now in Phase III trials. The principal aim of the modelling presented here is to explore the potential for current control measures to reduce parasite prevalence to a low level (defined here as below a threshold of 1% prevalence across all age groups detected through microscopy which represents a level below which surveillance would likely switch to case detection) as laid out in the control phase of the global elimination framework [45]. We illustrate our results by applying our model to six well-characterized transmission sites which represent the full range of transmission intensity–vector species combinations and seasonality patterns most commonly observed across Africa.

## Methods

### Simulation Model for Malaria Transmission Dynamics

**Figure 1:**
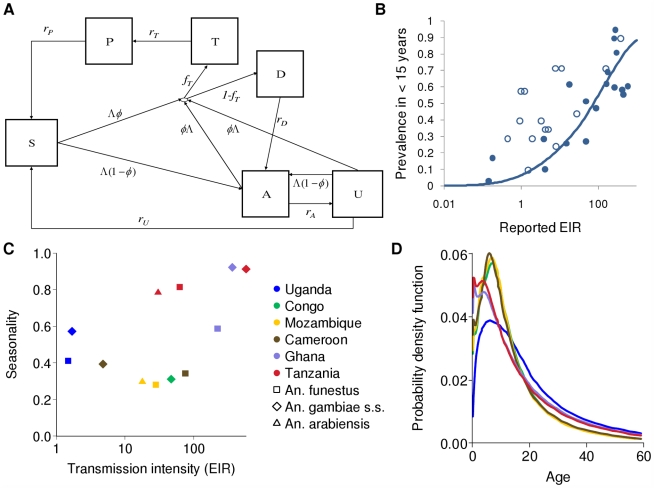
Transmission model; EIR, prevalence and seasonality; and infectious reservoir. (A) Flow diagram for the human component of the model. S, susceptible; T, treated clinical disease; D, untreated clinical disease; P, prophylaxis; A, asymptomatic patent infection; U, asymptomatic sub-patent infection. (B) The relationship between EIR and parasite prevalence in children under 15 y. Solid line: fitted relationship; filled circles: data representative of this age group; open circles: data from other age groups (mostly younger) used in the model fitting. (C) The relationship between transmission intensity characterized by EIR and seasonality, defined as the proportion of EIR over a single calendar year that occurs within the peak three months of transmission. The colours of the markers indicate the different transmission settings and the shapes the species. (D) The estimated age-specific infectious reservoir for the different transmission settings defined in (C), with the same colours as (C). This is defined as the product of the age-specific biting rate, age-specific prevalence states (T, D, A, and U), state-specific onward infectivity to mosquitoes and the size of the population at this age.

We developed a stochastic simulation model for *P. falciparum* transmission dynamics in which people are represented as individuals while vectors are represented as aggregated populations, stratified by species. The model builds on an earlier compartmental model which incorporates the acquisition and loss of immunity to disease and to detectable parasitaemia [46],[47], but is extended to incorporate infection-blocking immunity and heterogeneity in biting rates. Full technical details are given in Protocol S1 and the flow diagram is presented in Figure 1A. Briefly, individuals begin life susceptible (S) to infection but with partial maternal immunity determined by the level of immunity in women of childbearing age. Maternal immunity decays in the first six months of life, thereby increasing susceptibility to disease. Individuals become infected at a rate determined by the force of infection in the population (Λ), which is determined by the ratio of vectors to humans, the biting rate per mosquito on humans, the proportion of infectious mosquitoes in the vector population, and the person's level of anti-infection immunity. On infection, they pass through the liver (pre-patent) stage and then either develop clinical disease (with a probability *φ* determined by their current level of anti-disease immunity) or develop patent (detectable under microscopy) asymptomatic infection (1−*φ*). Those who develop clinical disease have a fixed probability (*f_T_*) of being treated successfully (T), in which case they will clear infection and, depending on the drug, enter (with rate *r_T_*) a period of prophylactic protection (P) before returning (*r_P_*) to being susceptible to new infection. Those who fail treatment (1−*f_T_*) are assumed to eventually clear disease (D) and become patently asymptomatic (A) with rate *r_D_*. From patent asymptomatic infection, individuals will eventually move to a sub-patent stage (U) which can be an important component of the infectious reservoir [48], at a rate (*r_A_*) that depends on their current level of anti-parasite immunity. Sub-patent infection is eventually cleared (*r_U_*) and individuals return to being fully susceptible. From all infected states, acquiring a new infection in the presence of an existing infection (superinfection) is possible. Rather than explicitly tracking mixed infections, we assume that the new infection dominates and thus individuals move to either the clinical disease or asymptomatic states dependent on their level of anti-disease immunity. Individuals become infectious to vectors, at differing rates, in the clinical disease, patent and sub-patent asymptomatic stages—the states that compose the *human infectious reservoir* (Figure 1D). Four types of human immunity are included and are modelled dynamically. Maternal immunity, which protects against clinical disease, is assumed to decay exponentially from birth. Anti-disease immunity, which reduces the probability of developing clinical symptoms on infection, and infection-blocking immunity, are both exposure-driven whilst anti-parasite immunity, in which individuals control parasite densities and thus leave the patent infection state more quickly, is assumed to develop with age, conditional on having been exposed.

Three *Anopheles* vector species (*An. gambiae s.s.*, *An. funestus*, and *An. arabiensis*) are modelled explicitly as the predominant vectors in the transmission sites that we consider. Vectors begin susceptible and on taking an infectious bite move into a latent state. From this they become infectious to humans, with infectivity determined by their human blood index (HBI) and biting rate and are assumed never to recover before death. Vector density is assumed to follow a seasonal pattern as determined by fitting an appropriate functional form to entomological data from the areas considered (see Table 1 and Protocol S4).

**Table 1:** Summary of the six malaria transmission settings considered here.

Country	Location	Population	Type of Transmission	Reported Annual EIR (ibppy)	Fitted Annual EIR (ibppy)	*Anopheles* Species Relative Abundance	Reference
Cameroon	Nkoteng	Rural	Moderate, perennial	94	81	72% *An. funestus*; 28% *An. gambiae s.s.*	[97]
Democratic Republic of Congo	Kinkole	Rural	Moderate, perennial	48	43	Nearly 100% *An. gambiae s.s.*	[98]
Ghana	Kassena-Nankana District	Rural	High, seasonal	630	586	60% *An. gambiae s.s.*; 40% *An. funestus*	[99]
Mozambique	Matola, Maputo	Coastal suburb of capital	Moderate, perennial	28	46	42% *An. arabiensis*; 46% *An. funestus* (additional 12% *An. coustani* are not considered here)	[100]
Tanzania	Matimbwa	Rural	High, seasonal	703	675	85% *An. gambiae s.s.*; 10% *An. funestus*; 5% *An. arabiensis*	[101]
Uganda	Kjenjojo Kasiina	Rural	Low	7	3	65% *An. gambiae s.s.*; 35% *An. funestus*	[102]

### Model Parameterization

**Figure 2:**
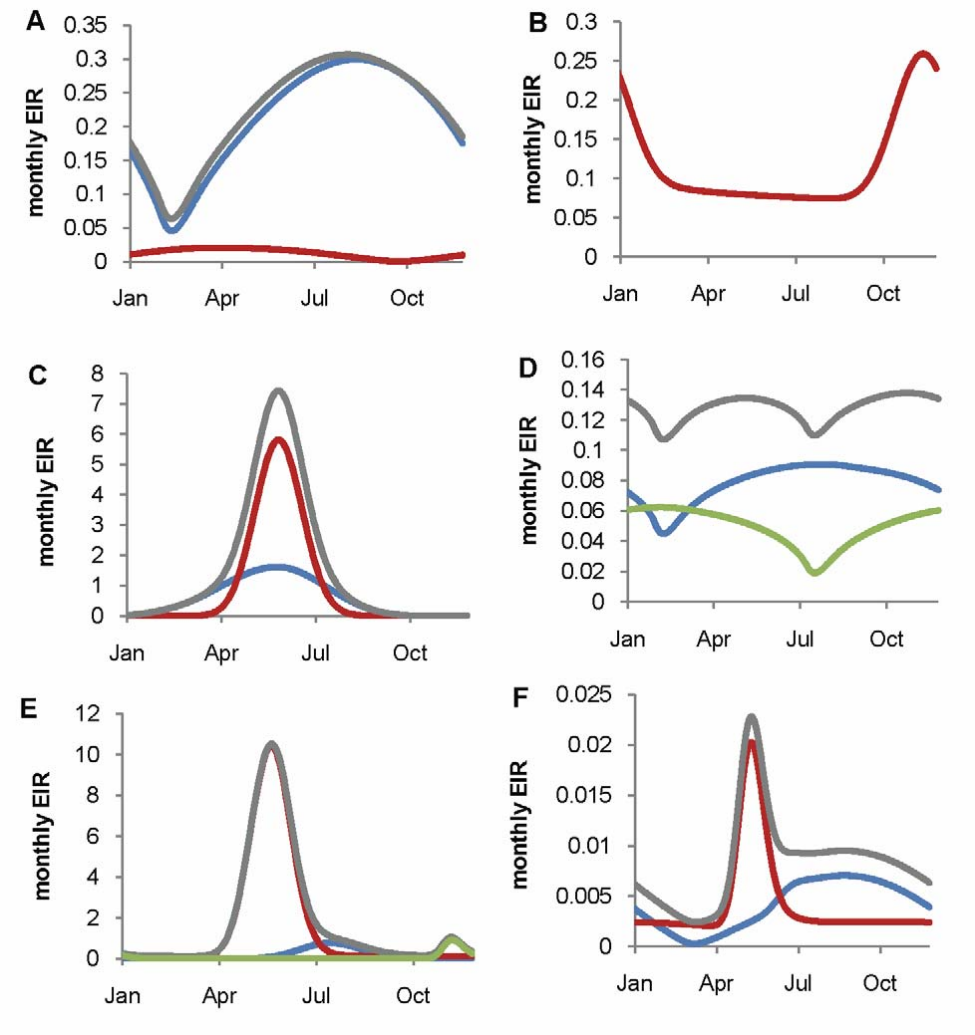
Fitted seasonal profile of EIR for the six transmission settings by vector species. The fitted seasonal profiles of EIR per day and fitted annual EIR were obtained by fitting a transformed sinusoidal function to reported time series of either EIR or mosquito densities in the settings (see Protocol S4). Grey, total; red, *An. gambiae s.s.*; blue, *An. funestus*; green, *An. arabiensis*. (A) Nkoteng, Cameroon; (B) Kinkole, DRC; (C) Kassena-Nankana District, Ghana; (D) Matola, Maputo, Mozambique; (E) Matimbwa, Tanzania; (F) Kjenjojo Kasiina, Uganda.

Model parameterization was undertaken in several stages. First, a literature search was undertaken to formulate prior distributions for all model parameters. Where there was no information in the literature, vague priors were used or parameters were fixed if they could not be identified from subsequent model fitting. The human model parameters were estimated by fitting the equilibrium model conditional on EIR using Bayesian Markov Chain Monte Carlo (MCMC) methods to data on the stationary distributions of parasite prevalence (by both microscopy and PCR) by age from 34 locations across a wide range of transmission intensities from Africa (see Protocol S3) and of clinical disease incidence from two settings in Senegal [49]. Site-specific prior distributions for EIR were used based on published data ([50] and Protocol S3). By fitting the model to these data we were able to characterize the relationship between EIR (ibppy, the number of infectious bites per person per year) and parasite prevalence (Figure 1B). The parameters determining the onward transmissibility of the human infectious stages (clinical disease, patent and sub-patent infection) to mosquitoes were obtained by model fitting to data from human feeding studies and the Garki project [7],[51]–[54]. These parameters combined with parasite prevalence determine the age profile of the infectious reservoir (Figure 1D) [55],[56]. Only age-targeted strategies are sensitive to this profile. Parameters for the vector model were taken from the literature. A full listing of model parameters, their prior and posterior medians, and literature sources are given in Table S3.1 in Protocol S3. To run the model in specific settings, data on vector species composition, their seasonal profile, and the intensity of transmission (EIR) were extracted from the literature (Table 1, Figure 1C, Figure 2, and Protocol S4). A functional form was fitted to monthly data on either EIR or vector density to enable a single seasonal driver input (emergence of vectors) into the model. Full details of the settings and the seasonal profile fitting are in Protocol S4.

### Interventions

The implementation of each intervention is described briefly below. Full mathematical details and tables of parameter values are provided in Protocols S2 and S3.

#### Long-lasting insecticide-treated nets

We adapted an existing model [36] to our individual-based framework. Nets are assumed to have four effects: direct killing of a mosquito that lands on them, repellency which results in a longer gonotrophic cycle and possible diversion to a non-human blood host, a direct protective effect for the individual sleeping under the net, and a reduction in transmission from infected individuals sleeping under the net to susceptible mosquitoes. The degree of indoor-biting (endophagic) behaviour for the different species is incorporated into the model when assessing the LLIN effect. These behaviours are assumed to remain constant throughout the intervention.

#### Indoor residual spraying

IRS was added to the LLIN model as an additional intervention which can kill mosquitoes as they rest within the house or repel them before they feed. In the model the repellency effect extends the duration of the gonotrophic cycle in the same way as the repellency effect of LLINs. For IRS the killing effect depends primarily on the indoor-resting (endophilic) nature of the species as well as its HBI. Simulations assumed a DDT-like insecticide with a half-life of 6 mo which acts by repelling and killing mosquitoes [57].

#### Switch to ACT as first-line treatment

Effective treatment (i.e., treatment which fully clears infection) was assumed to be given to a proportion of those developing clinical disease. Treatment failures were not explicitly modelled but are assumed to follow the same infection path as untreated infections. The half-life of the drugs pre-ACT (where we assume sulphadoxine-pyrimethamine [SP] was first-line therapy) and following ACT introduction determine the period of prophylaxis. In addition, the gametocytocidal effect of ACTs was incorporated as a reduction in onward infectiousness as in a previous model, based on data from human-to-mosquito transmission experiments involving treated patients [27].

#### Mass drug administration

We considered the impact of a mass screening and treatment approach (MSAT) using a single dose of an ACT. We assumed that a rapid diagnostic test (RDT) would have approximately the same sensitivity as microscopy and thus all those in the clinical disease or asymptomatic patent infection stages would receive the drug, but that the uninfected and sub-patent infected individuals would not. The ACT was assumed to clear any infection present and provide a period of prophylactic protection (25 d, corresponding to an artemisinin coupled with a drug such as SP). The coverage level refers to the number of individuals screened.

#### Pre-erythrocytic vaccine

A pre-erythroyctic vaccine was assumed to reduce the probability of transmission from mosquitoes to people. It remains unclear whether this lower exposure to infection will affect the development of anti-disease immunity. Here we assume that it does but that it has no effect on the development of anti-infection immunity. Individual vaccine efficacy was assumed to decay exponentially with a half-life of 3 y. The vaccine is delivered through the Expanded Program for Immunization (EPI) and given at ages 3–5 mo, or as a mass vaccination program across all ages every 3 y.

### Transmission Settings

We considered the impact of these interventions, individually and in combination, in six different settings that characterize the spectrum of transmission patterns of *P. falciparum* across Africa. These settings range in transmission intensity from measured EIRs of approximately 5 to over 500, translating in our model to parasite prevalence in 2- to 10-year-olds of 14% to 85%. In 2007, 80% of Africa's population was estimated to reside in an area with parasite prevalence in 2- to 10-year-olds of >5% and 50% in an area with prevalence >40% [16]. These specific settings, summarized in Table 1, Figure 1C, and Figure 2, were chosen because of the large number of both entomological and clinical studies undertaken in these areas and to represent patterns of perennial/seasonal transmission of varying intensity and with different mixes of *Anopheles* species. We fitted the model to data from these settings, which we take as our baseline scenarios.

For each scenario, we present the mean of ten simulation runs in a population of 10,000 individuals, which was sufficient to approximate the dynamics in a larger population. The population size was assumed to be static over time, with age structure based on data from Tanzania. After introducing infection, the model was run for 50 y to reach equilibrium representing the situation in the year 2000. Between 2000 and 2010 we increased the distribution of LLINs from a baseline of zero coverage to a maximum of 20% coverage [58] and implemented a switch to ACT as first-line therapy in the year 2000. Combinations of interventions were then introduced from 2010 onwards. Note that this does not necessarily reflect the true intervention programs in place in these settings in these years, and hence model outputs do not directly predict expected patterns in these settings; rather they give an indication of the likely effectiveness of the modelled intervention packages in different setting types.

### Intervention Package Scenarios

**Figure 3:**
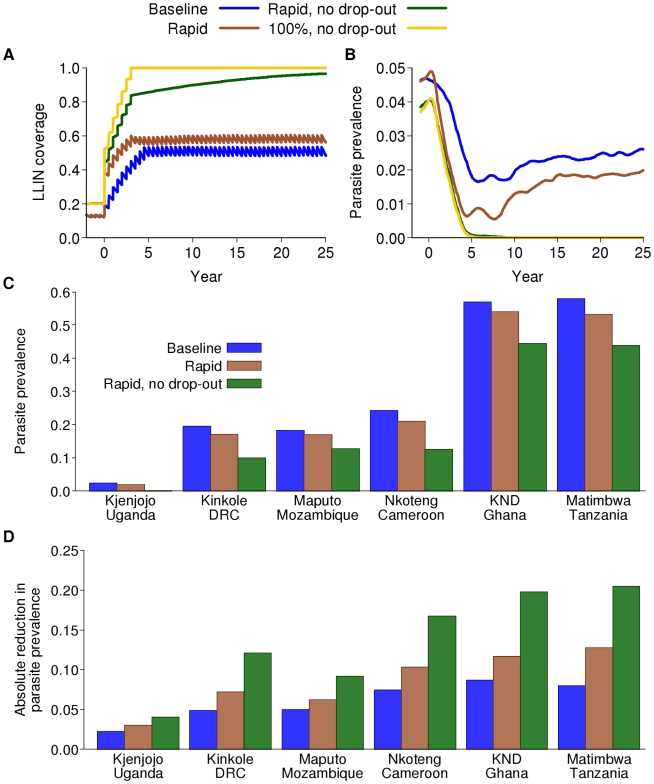
Impact on parasite prevalence of LLINs alone. (A) Example of the ways in which coverage of LLINs is considered to increase in various model scenarios. Baseline (blue): our baseline scenario in which 80% coverage is achieved over five years but adherence also decays between net distribution rounds; rapid (brown): as baseline but with more rapid scale-up to 80% coverage; rapid, no drop-out (green): rapid scale-up to 80% coverage with no decay in adherence; 100% (yellow): 100% coverage with no decay in adherence (theoretical maximum effect). (B) Model-predicted impact on parasite prevalence over calendar time of four scenarios for LLIN scale-up combined with an earlier switch to ACT as first-line therapy in Kjenjojo Kasiina, Uganda. (C) Final parasite prevalence and (D) absolute reduction in parasite prevalence after 15 years of a sustained intervention program in the six transmission settings with the baseline scenario for LLIN distribution, the rapid scenario for LLIN distribution, and the rapid scenario with no loss of adherence for LLIN distribution.

Coverage here is defined as the proportion of individuals receiving an intervention (for LLINs ownership, for vaccination those receiving the vaccine, for IRS those that reside in houses where spraying occurs, and for MSAT the number of individuals screened). We separately consider the impact of adherence/usage for LLINs, which is assumed to decay over time. This proportion of people using LLINs is termed effective coverage. For IRS we assume no loss of adherence. For MSAT we assume that all those who are screened and positive on microscopy take the drug. Similarly, for the vaccine we assume that those offered it accept. Finally, for all interventions there is a decay in protective efficacy over time for those who have received and use the intervention. For LLINs this is due to wear-and-tear and loss of insecticidal effect. For IRS we model the loss of insecticidal effect. For vaccines we assume that efficacy declines through waning protection. Unless stated otherwise we assumed that IRS and MSAT were given at 80% coverage (the maximum achievable in well-managed control programs [59]) and the vaccine at 90% coverage (based on EPI distribution statistics). For the roll-out of LLINs we considered two realistic scenarios. In the first, distribution was increased gradually to a maximum of 80% within 5 y and a new net was distributed to individuals every 5 y. In the second, we assumed almost immediate distribution at 80% coverage, redistribution every 5 y, plus delivery of a net to 80% of newborn infants and an average of 0.75 adults for every infant who receives a net (Figure 3A). These coverage levels are similar to the targets set for 2010 for scaling up for impact in the Global Malaria Action Plan [18]. In both scenarios, we assume that LLIN use wanes over time so that effective coverage is lower. Here we assumed an exponential decay at a rate 0.2 per year so that after 5 y effective coverage is approximately 37% of the baseline level. We also considered the impact of a theoretical (unachievable) maximum of 100% coverage with LLINs coupled with no decay in usage over time (Figure 3A). Protective efficacy of the nets due to decaying insecticide efficacy and wear-and-tear was assumed to decay exponentially with a half-life of 2.64 y (Protocol S3 and [60]). We did not consider any decay in effective coverage of IRS as we assumed that coverage remained constant at each round (i.e., people do not refuse to have their house sprayed as the intervention goes on). The protective efficacy of DDT was assumed to decay exponentially with a half-life of 6 mo (Protocol S3 and [57]). Adherence to LLINs given receipt was assumed to be independent of IRS acceptance.

We undertook preliminary runs for IRS and MSAT to identify the optimal time of year for annual programs. The optimal time was defined on the basis of providing the maximum reduction in mean prevalence of parasitaemia across all age groups in year 10 of the intervention campaign. We found that in those settings which have a clear seasonal peak in the EIR, it is always optimal to spray just before the upward trend in EIR. In settings with less seasonality, there is less difference in impact, but spraying at the start of the main transmission season tends to remain optimal. In contrast, across most settings, the optimal time of year to mass treat in terms of reducing overall prevalence of asexual parasitaemia as an endpoint is at the beginning of the period of lowest EIR (also shown in [61]), which generally occurs approximately 2 mo after peak slide prevalence. For scenarios in which IRS and MSAT were undertaken every 6 mo, they were implemented at the optimal time of year as defined above, plus 6 mo thereafter.

Effective coverage and protective efficacy do not alone determine intervention effectiveness, as they also depend on whether the same individuals receive multiple interventions or whether interventions are randomly distributed across the population. We therefore allowed correlations between repeat distribution for each individual intervention (where a correlation of 0 means that redistribution is completely random and of 1 that redistribution always occurs to those who had previously received the intervention). We also allowed correlations between receiving LLINs, IRS, and MSAT. Here a positive correlation means that individuals who receive one intervention are also more likely to receive the other (which could reflect access to interventions) whilst a negative correlation means that those who receive one intervention tend not to receive the other (which would reflect a propensity not to use multiple interventions).

As our focus is to consider intervention packages aimed at reducing transmission, our primary outcome was the annual mean prevalence of asexual parasitaemia as measured by microscopy in the whole population up to 25 y following the start of the intervention program. We chose this rather than prevalence restricted to children as it enables us to correctly compare age-targeted interventions. We specifically do not focus on short-term “predictions” or timelines, as our sensitivity analysis shows that these are highly dependent on parameters relating to the loss of acquired immunity (which impact the fitted duration of infection). Currently these parameters are not well-estimated from the available data (see Section 5.2.1 in Protocol S5, and Box 1). Furthermore, time scales of impact will inevitably depend on the speed with which scale-up of interventions occurs and so cannot be reliably predicted without detailed assessment of local situations.

**Box 1. Uncertain Parameters**

Whilst models can be useful tools in setting realistic expectations for intervention programs, some key parameters in our current model are based on limited data. Further empirical work in these areas could improve future models. These include:

- The duration of natural infection and the extent to which super-infection prolongs this duration or increases infectivity;
- The rate of acquisition of immunity at different transmission intensities, and the rate of loss of immunity when transmission is reduced;
- The bionomics of the principal vector species and the impact of vector-targeted interventions on them;
- Detailed data on the speed with which coverage of interventions is scaled up, heterogeneity in coverage levels achieved, and the degree of adherence to the interventions over time.

### Software

A user-friendly software package for reproducing the simulations presented here, as well as other potential combinations of the interventions included in this paper, is freely available to download from our Web site (http://www1.imperial.ac.uk/medicine/about/divisions/publichealth/ide/research_groups/malaria/). A short summary of the interface is provided in Protocol S6.

## Results

### Continued Scale-Up of LLINs

Continued scale-up of LLINs from the baseline assumption of 20% coverage could potentially reduce transmission across all six transmission settings, given that the dominant vector species in these settings are primarily endophagic and their peak biting times coincide closely with normal sleeping hours [62] (provided changes in mosquito behaviour in response to the interventions are not dramatic). However, the magnitude of the effect will depend not only on the intensity of transmission in each setting but also how roll-out is achieved, the final level of coverage, adherence to LLIN use, and the decay in insecticide effectiveness over time. Figure 3A shows four potential scenarios for scale-up if nets are redistributed every 5 y. Theoretically, the greatest impact is achieved with rapid deployment, 100% coverage, and perfect adherence. However, even at this unrealistically high level, the efficacy will be less than its maximum due to decaying effectiveness of the insecticide. Even at the target coverage levels of 80%, with gradual roll-out and realistic adherence, effective coverage levels can, on average, be as low as 50% (Figure 3A). The additional decay in insecticide efficacy over time can result in protective coverage levels as low as 30%. This is even without the additional limitation of an interrupted supply chain, which is likely to reduce effective coverage further [63].

In the low-transmission setting of Kjenjojo Kasiina, Uganda, the basic reproduction number (*R*_0_) is already close to 1 in the absence of additional interventions. Thus, parasite prevalence can be reduced to below the 1% threshold over a 15 y time horizon with LLIN use alone (Figure 3B). However, even in this relatively low-transmission setting, high levels of coverage and adherence are required. Furthermore, with decaying adherence in their use it is likely that transmission will be sustained, albeit at a low level. Furthermore, if LLINs have a lower killing effect than that assumed here, our model would predict sustained transmission in this setting (Figure S5.6 in Protocol S5).

In contrast, in the moderate-transmission settings of Nkoteng (Cameroon), Kinkole (Democratic Republic of Congo or DRC), and Maputo (Mozambique), and in the high-transmission settings of Kassena Nankana District (KND) (Ghana), and Matimbwa (Tanzania), scale-up of LLINs alone does not reduce parasite prevalence to below 1%, even over longer time periods (Figure 3C). We can, however, expect to see dramatic declines in the first five years of the program followed by an increase to new endemic levels as levels of immunity in the population change (Figure S5.1 in Protocol S5). The time scale of this rebound is difficult to ascertain from current data due to uncertainty in the rate of loss of acquired immunity (see section 5.2.1 in Protocol S5, and Box 1).

In high-transmission settings, with continued scale-up of LLINs to 80% coverage within five years, the absolute drop in prevalence is between 5% and 10%. If rapid scale-up occurs and adherence is sustained, drops in prevalence of 20%–25% can be expected (Figure 3D). However, despite the smallest relative impact occurring in the high-transmission settings, because most cases of infection and disease occur in these settings, the absolute impact in terms of numbers of infections averted will be greater. Thus, in terms of reduction in infections per net distributed, impact will be greatest in these high-transmission settings.

### Additional Use of IRS and MSAT

**Figure 4:**
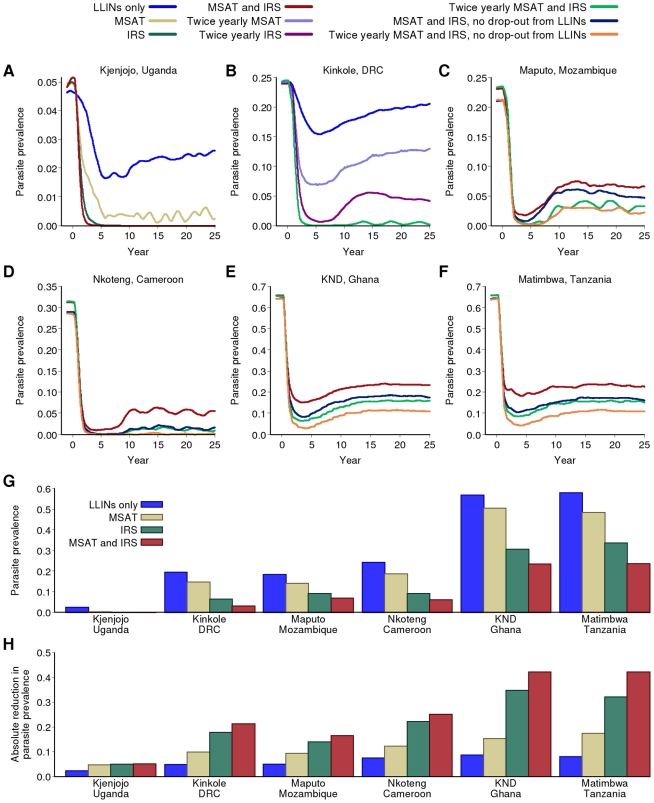
Impact of combining LLINs with IRS and MSAT. (A–F) Impact of intervention scenarios incorporating IRS and MSAT on parasite prevalence in the six transmission settings. All scenarios include the earlier switch to ACT as first-line therapy. “LLIN only” uses the baseline scale-up for coverage. All other scenarios include LLIN scale-up using the baseline scenario except where noted. (G and H) Final parasite prevalence and absolute reduction in prevalence after 15 years of a sustained intervention program in the six transmission settings with baseline scenario for LLIN distribution; baseline LLIN + yearly MSAT; baseline LLIN + yearly IRS; baseline LLIN + yearly MSAT + yearly IRS.

Whilst continued scale-up of LLINs is predicted to reduce transmission substantially, under realistic assumptions about the level of coverage and adherence to LLIN use, additional tools will be necessary in many settings. In Kjenjojo Kasiina, Uganda, yearly rounds of IRS with DDT combined with continued scale-up of LLINs to 80% coverage is predicted to locally eliminate transmission (Figure 4A). Yearly rounds of MSAT as an alternative to IRS tend to have less impact although this would also achieve a reduction below the 1% parasite prevalence threshold.

In the moderate-transmission setting of Kinkole, DRC, more intensive rounds are required. Thus, in this setting, twice yearly IRS and MSAT are required to reduce parasite prevalence below the 1% threshold (Figure 4B). In contrast, in the slightly higher-transmission setting of Nkoteng, Cameroon, this is not sufficient in itself and additional faster scale-up of LLINs is needed to achieve this threshold (Figure 4D). In Maputo, Mozambique, in which transmission intensity as measured by EIR is similar to Kinkole, DRC and lower than Nkoteng, Cameroon, even these more intense programs are unable to reduce prevalence below the 1% threshold (Figure 4C). This is due to the high proportion of transmission that occurs via *An. arabiensis* in this setting, whose more exophilic behaviour reduces the impact of IRS on transmission. Assuming a lower degree of exophilic behaviour of this species compared to our baseline assumption, this conclusion continues to hold (section 5.2.2 in Protocol S5). In all three moderate-transmission settings, IRS with an insecticide similar to lambdacyhalothrin (which is less repellent and hence more lethal but has a shorter half-life than DDT) is predicted to have a lesser effect on transmission than DDT (Figure S5.7 in Protocol S5).

In both high-transmission settings (KND, Ghana and Matimbwa, Tanzania), current tools are insufficient to reduce parasite prevalence below the 1% threshold (Figure 4E and 4F; see also higher levels of adherence and coverage in Figure S5.3 in Protocol S5, and higher frequency of MSAT in Figure S5.8 in Protocol S5). However, in both settings, an intense program involving rapid scale-up of LLINs with sustained adherence and twice-yearly rounds of MSAT and IRS could result in marked declines in prevalence from 60% to 10% in the population as a whole (Figure 4E and 4F). However, in these settings, the interventions would need to be sustained indefinitely to maintain this new endemic level. Yearly IRS and MSAT combined with 80% coverage of LLINs is predicted to reduce parasite prevalence after 15 y to below 10% in moderate transmission settings and below 25% in high-transmission settings (Figure 4G). Again, the absolute reduction will be greatest in the latter, with a 40%–50% drop in parasite prevalence in these settings (Figure 4H).

### Targeting and Overlap in Intervention Coverage

**Figure 5:**
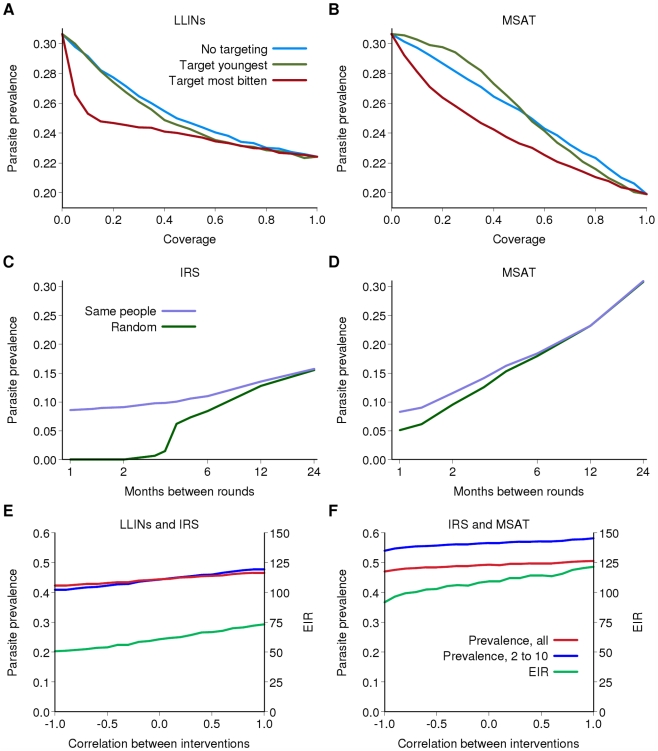
The effect of non-random distribution of interventions. (A and B) Parasite prevalence after 15 years of an intervention program as a function of the target coverage of (A) LLIN distribution and (B) MSAT for Kinkole, DRC. Blue: if the intervention is distributed randomly; green: if the intervention is preferentially distributed to the youngest children; red: if the intervention is preferentially distributed to those who are bitten most frequently (excluding age dependency in biting rates). (C and D) Parasite prevalence after 15 years of a single intervention program as a function of the frequency of the intervention and whether successive rounds are given randomly (green) or to the same people (purple) for Kinkole, DRC. (C) IRS; (D) MSAT. (E and F) Parasite prevalence in all individuals (red), in 2- to 10-year-olds (blue) and EIR (green) after 15 years of a combined intervention program as a function of the correlation in receipt of the two interventions for KND, Ghana. A correlation of 0 represents random distribution at each round, 1 represents those receiving one intervention also receive the other and −1 represents those receiving one intervention do not receive the other. (E) IRS and LLIN; (F) IRS and MSAT. For (E) and (F) there is 50% coverage per round for IRS and MSAT and the baseline scenario for LLINs.

LLIN distribution programs initially focused on young children as one of the high-risk groups for developing severe disease. However, as shown in Figure 5A and elsewhere [23],[24], this strategy is unlikely to have an additional impact on transmission, because the youngest children tend not to be major contributors to the infectious reservoir (Figure 1D). However, if limited coverage is achievable, substantially greater reductions in prevalence could be obtained if, for a given level of distribution, nets were targeted towards those living in the local foci of transmission which impact strongly on sustaining transmission [38],[42],[43],[64]. Thus in Kinkole, DRC, in a program of LLIN distribution with a low 20% coverage, if distribution is prioritised to those at highest risk we could expect a reduction in prevalence after 15 y of approximately 6% compared to a reduction of 3% if the same number of nets were distributed randomly. A similar picture emerges for MSAT programs (Figure 5B), although the effect of targeting is greater for LLINs because in addition to their direct protective effect, they kill mosquitoes in proportion to the rate at which the protected person would have been bitten.

With any intervention, it is likely that the same individuals or villages will tend to access the intervention at each distribution round. Thus for example, if 80% coverage of LLINs is achieved, but at each redistribution the same 80% receive the intervention, then after three rounds of redistribution the percentage of the population ever receiving an LLIN is 80%. However, if this 80% coverage reflects random distribution, then after three rounds the percentage of the population ever receiving an LLIN is 100×(1−0.2×0.2×0.2) = 99.2%. Figure 5C and 5D shows the predicted effect of rounds of IRS and MSAT between these two extreme (systematic versus random coverage) scenarios. In both cases, assuming random distribution results in an overestimate of the effect of the intervention, and this difference increases the more frequently IRS or MSAT is undertaken. Thus, to optimize program effectiveness it is necessary to ensure that as wide a proportion of the target population is reached by the intervention.

In addition to correlations between those who receive an individual intervention, there is likely to be overlap in those who are offered different interventions. This is likely to be most strongly correlated for IRS and LLINs, given the perception of these interventions as providing direct protection to the individual or household. A positive correlation will occur if the same individuals access the interventions. Under these scenarios, we can expect the least impact of the intervention program (Figure 5E). However, if uptake is negatively correlated, for example if those who are offered IRS and LLINs choose only to have one, for the same overall coverage levels of the individual interventions total population coverage is increased over and above naïve expectations assuming both are randomly distributed. This increased total coverage results in the largest reductions in transmission (Figure 5E). Similar effects are observed for IRS and MSAT, although again, this is not as pronounced as for LLINs given that there is less redundancy between IRS and MSAT than between two antivectorial measures (Figure 5F).

### Additional Impact of RTS,S/AS01 Vaccine

**Figure 6:**
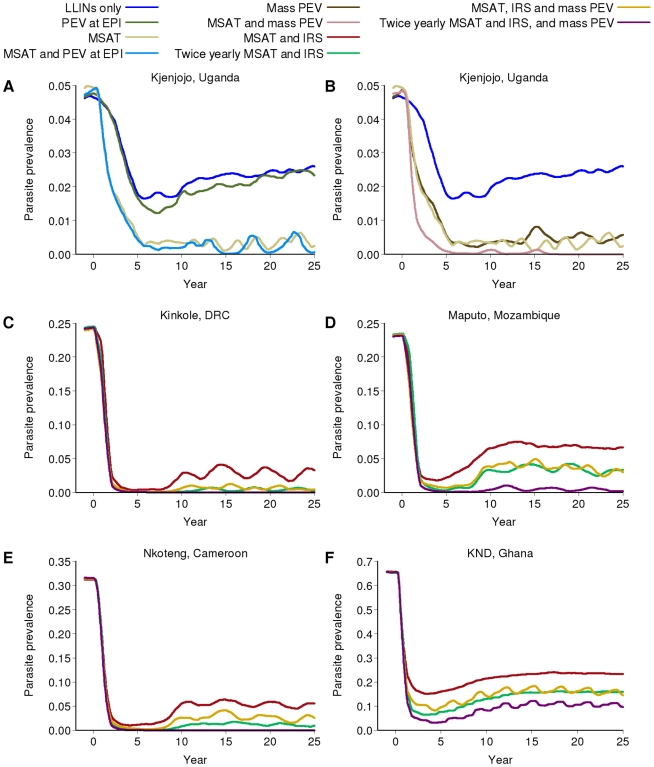
Impact of additional vaccination on parasite prevalence in the different transmission settings. All runs assume the RTS,S vaccine is 50% efficacious and has a half-life of 3 years. PEV at EPI denotes the pre-erythrocytic vaccine being given through the Expanded Program on Immunization, whilst mass PEV denotes a mass vaccination campaign. All runs include LLINs. (A) PEV at EPI with or without additional MSAT in Kjenjojo, Uganda (B) Mass PEV with or without additional MSAT in Kjenjojo, Uganda (C to F) MSAT and IRS with mass PEV in: (C) Kinkole, DRC, (D) Maputo, Mozambique, (E) Nkoteng, Cameroon and (F) KND, Ghana.

In the low-transmission setting of Kjenjojo Kasiina, Uganda, RTS,S (when it becomes available) could further reduce transmission and thus negate the need for additional rounds of IRS to speed declines. As found by others [65],[66], vaccination at birth under the EPI is expected to have relatively little impact either with or without additional rounds of MSAT (Figure 6A). If mass vaccination every 3 y is undertaken as an alternative alongside the baseline scale-up of LLINs to 80% coverage, prevalence is predicted to fall to under 1%.

In the moderate transmission settings of Kinkole, DRC (Figure 6C), Maputo, Mozambique (Figure 6D), and Nkoteng, Cameroon (Figure 6E), continuation of programs incorporating IRS and MSAT in addition to LLIN distribution will be needed even if a vaccine is available. However, with a mass vaccination program prevalence in all three sites can be driven below 5%. In Maputo especially, where IRS is predicted to be less effective, an additional vaccination program has a noticeable further impact on prevalence. In both high transmission settings (KND, Ghana, Figure 6F; and Matimbwa, Tanzania, results not shown), mass vaccination results in modest reductions in prevalence. Across all transmission settings, a more efficacious vaccine with a longer duration of protection would further reduce transmission (section 5.2.6 in Protocol S5).

## Discussion

If deployed in combination, current interventions can result in substantial declines in malaria prevalence across a wide range of transmission settings. Our results show that in areas with relatively low transmission (EIR<10 ibppy), increased distribution and use of LLINs, coupled with the switch to an effective ACT as first-line therapy, could reduce transmission to very low levels if high levels of coverage and adherence are achieved. Defining low-transmission areas as those where parasite prevalence in 2- to 10-year-olds is under 25%, approximately 20%–50% of individuals living in areas of stable risk of *P. falciparum* transmission in Africa live in such settings [16]. Additional use of IRS and/or MSAT in these settings would speed this reduction and also allow overall parasite prevalence to be reduced to <1% even if adherence to LLIN use is not perfect. These results agree with recent observations made in a very low transmission setting in Western Kenya, in which the parasite appears to have been eliminated in an area in which ACT and LLIN usage have been coupled with IRS rounds [67]. Large reductions have also been achieved in Zanzibar, where the preintervention parasite prevalence was 9% in children aged 0 to 5 y and 12.9% in children aged 6 to 14 y [13]. After a switch to ACT as first-line therapy and high coverage of both LLINs and IRS rounds from 2003, parasite prevalence in all age groups is now well below the 1% threshold. The challenge in such settings is to sustain interventions at a sufficient level to maintain effective control in the face of reintroduction from neighbouring areas via human migration and travel.

In some moderate-transmission settings it is also possible to reduce parasite prevalence below the 1% threshold with existing tools. In our example settings, this could be achieved in Kinkole, DRC where the endemic EIR was 48 ibppy if an intensive program of twice-yearly IRS and MSAT were combined with increasing LLIN coverage to 80% levels. In the slightly higher transmission setting of Nkoteng, Cameroon (EIR = 96 ibppy), current tools could reduce transmission below the 1% threshold but in this case (perhaps unrealistically) high levels of adherence to LLIN use would also be needed. Thus the first phase of elimination programs is achievable in many areas in which the LLIN and IRS in combination are effective (that is, in areas with primarily endophilic vectors). Additional use of MSAT, to date not considered by many programs, has the potential to speed further declines in prevalence.

We considered one area, Maputo, Mozambique, in which the high proportion of *An. arabiensis* (exhibiting a high degree of exophilic behaviour), made elimination more difficult. Whilst the scale of the declines that our model predicts are similar to those observed in an IRS-based campaign in that area (that commenced in 2000 using Bendiocarb rather than DDT [4]), this study also demonstrated a greater impact on the population of *An. arabiensis* compared to that resulting from our model. This may be because our estimate of the degree of exophilic behaviour is too high (see Protocol S3) or because mosquito behaviour changes both with season and with setting [35],[68]–[71] and requires further exploration.

In high transmission intensity settings, current tools can be used to substantially reduce transmission and the associated disease burden, but are insufficient to drive prevalence below the pre-elimination threshold. This finding is not surprising given the high basic reproduction numbers previously estimated in large parts of sub-Saharan Africa [43]. Such outcomes have been observed in the Bioko Island control program where, with intensive ongoing interventions, parasite prevalence in 2- to 5-year-olds fell from 42% to 18% between 2004 and 2008 [14]. Similarly, in the 1970s Garki project in Nigeria, an area of moderate to high transmission (annual EIRs in the range 20–130 ibppy), substantial declines in prevalence were recorded but elimination was not achieved [7]. In these settings, additional new tools are likely to be required if pre-elimination targets are to be achieved.

Whilst a detailed comparison of the range of potential tools under development is beyond the scope of this paper, there are two broad areas of innovation that merit further consideration. The first aims to target the mosquitoes that are not reached by current interventions, particularly those on whom indoor-targeted interventions are least successful. Notably, this includes major species such as *An. arabiensis*, which preferentially rest outdoors after feeding and may also obtain blood meals from non-human animals. These mosquitoes could be targeted in a number of ways, including additional interventions that are applied on non-human hosts [72], killing adult females feeding or resting outdoors [73]–[75], or at source in the larval habitat [76],[77]. Secondly, our results on the levels of human adherence required in high-transmission settings suggest that interventions that do not strongly depend on human participation are likely to be needed. The methods outlined above are examples of such approaches.

Our results confirm findings by others that the bionomics of the local vector species, including the degree of exophagy, exophily, and zoophagy [35],[36],[78], can potentially be a strong determinant of intervention success. Current tools, in particular LLINs and IRS, are focused towards species with strong endophagic, endophilic, and anthropophagic tendencies. Further data on the degree of endophilic behaviour of the different *Anopheles* species, coupled with information on how these parameters may change in response to interventions (we assumed here that they remain fixed),are critically needed to understand the longer-term impact of IRS and LLINs on transmission. Historically, there is some evidence of species replacement following the introduction of IRS in three different geographical locations [79]. More recently, a shift in species relative abundance (though not replacement or increased density) has been observed in Western Kenya following high coverage of LLINs [80],[81]. In addition, mapping of vector species distribution and proportional composition [17] is critical to the ability to predict program success outside of the well-studied research areas.

Behavioural aspects of intervention programs are characterized in multiple ways. For example, the WHO report bed-net coverage as the number of nets distributed per person at risk [5], whilst Malaria Indictor Surveys collect data on the proportion of households owning a net or sleeping under a net [82]. Our results demonstrate that patterns of coverage and effective coverage are an important determinant of intervention success and may be one reason why simple models of LLIN impact have tended to appear highly optimistic [35]–[38]. Furthermore, it is unrealistic to assume perfect and uniform adherence. Indeed, rates of sleeping under LLINs tends to be highest in young children, but lower in school-aged children [83], who are important contributors to the infectious reservoir (Figure 1). Furthermore, whilst we did not explicitly consider reduction in adherence or take-up of IRS, this is likely to occur after repeated rounds as perceived risk declines, and will reduce the impact of the intervention. Receipt of interventions is also an important consideration in assessing impact, particularly if coverage levels are low. It is well recognized that malaria transmission is highly focal with some individuals at much higher risk than others [42],[43],[64],[84],[85]. Our results confirm other models' findings [43],[64] that, by targeting interventions at areas of intense transmission, substantially greater reductions in transmission are possible than by distributing them randomly or by focusing distribution towards younger children. However, little attention has previously been paid to the heterogeneous distribution of interventions within such target populations. In general, the impact of an intervention will be lower if the same individuals in the target population continually receive and adhere to the intervention than if distribution fully covers the target population. Thus data on repeat uptake of interventions would be useful to determine true target population coverage levels. Furthermore, health systems will need to be strengthened and laboratory capacity put in place to allow rapid identification of these foci. In addition, overall coverage levels can potentially be enhanced through consideration of a wide range of different delivery mechanisms appropriate to the local setting [86]–[88].

One aspect with the potential to hinder elimination campaigns not considered here is the development of resistance—either to drugs, to the insecticides used to treat nets or for indoor residual spraying, or to vaccines—and the potential for alterations in the behaviour of the vector in response to the interventions. Resistance to DDT was a particular problem during the GMEP and is credited with being a major reason for the abandonment of the program. DDT resistance at varying levels has now been reported in over 50 anopheline species [89]; thus, to reduce the further emergence of resistance, elimination campaigns should aim to reduce transmission as rapidly as possible. The recent emergence of partial drug resistance to artemisinin in Cambodia [90] has further highlighted the need to guard against and reduce the emergence and spread of resistance, particularly as access to treatment is scaled up.

Our model is necessarily a simplification of the more complex dynamics underlying malaria transmission and control, so numerical results should be interpreted more as providing intuitive insight into potential scenarios than as firm predictions of what might happen in a given setting. Furthermore, whilst we give an indication of impact over a 25-year time horizon (including graphs that track expected trends over this period), given the uncertainty in some of the key parameters, it is not possible to give short-term indications of impact or timelines. Precise, accurate prediction remains challenging for a number of reasons. First, the mean duration of asymptomatic infection, and the dynamics of acquisition and loss of immunity, are key parameters determining the speed of decline in parasite prevalence once transmission is reduced [47]. These are both poorly understood in semi-immune populations. These parameters also determine the time scale for which interventions would need to remain in place to ensure that a rebound in infection and disease does not occur. Current best estimates of model parameters suggest that this is likely to be decades rather than months or years, but further data are needed to refine these estimates.

Second, there are multiple model structures that can reproduce important characteristics of malaria epidemiology such as the age patterns of infection prevalence across different transmission settings. Whilst we have invested substantial effort in developing a modern statistical framework to better choose between model structures and to estimate associated model parameters, there are limited data to distinguish some aspects of the model. In the current exercise, we have focused on fitting the human model cycle to a wide range of datasets. This will be extended in future applications to fit the full cycle using explicitly seasonal models to more detailed data from specific research sites. In addition, the individual intervention models have not to date been validated by fitting to specific trial data. This process is underway. Such fitting will enable the addition of uncertainty bounds to model output through sampling of parameter posterior densities [91]. If feasible, this could be extended to incorporate model uncertainty using a Bayesian methodology [92].

Third, in our current model we use a relatively simple vector cycle in which the vector population is driven by a constant birth rate. This may underestimate the additional impact of interventions that increase vector mortality and thus reduce population-level fecundity. Vector models which incorporate capacity constraints and behavioural change are a natural extension that may better represent competition for larval habitats [93]. However, to date, such models have not been adequately validated against weather measurements and entomological data and thus further work is required to obtain a model that can reproduce entomological patterns from multiple transmission settings.

Last, our current model has been developed and parameterized to be applied to single locations. It thus considers isolated areas and does not address the focal and heterogeneous nature of transmission on a wider spatial scale or the connectedness of local populations. As such, the current model cannot be used to assess the risk of reintroduction of the parasite from outside areas, which has been shown to be a major challenge in ongoing control [94]. However, it is possible to extend this framework to a fully spatial continental-scale simulator. The major challenge here is not in developing the software tool but in parameterising the model across settings. Basic requirements of such a model, e.g. human population size in each area, are not well known across parts of Africa, although synthetic data derived from satellite observations can be used as a proxy [95],[96]. In addition, such models require local-level information on vector species, seasonality patterns, intensity of transmission, and human movements to enable assessment of the risks of transmission spatially.

Despite these limitations, mathematical models based on the biology of the transmission cycle provide an appropriate tool for a range of stakeholders to explore the potential impact of current and future interventions on malaria transmission and disease burden in a systematic manner. Further development of the models and approaches outlined here can help to identify optimal policies for the range of stages of malaria elimination programs from the consolidation phase outlined here, through the pre-elimination and elimination phases, to sustained elimination. By considering current tools and exploring potential future interventions, models can help us to understand the limits of current strategies and evaluate the potential for future products to achieve the ultimate goal of global eradication.

## Supporting Information

Alternative Language Abstract S1 Abstract translated into French by Emilie Pothin.
(0.03 MB DOC)

Alternative Language Abstract S2 Abstract translated into Spanish by MGB.
(0.03 MB DOC)

Alternative Language Abstract S3 Abstract translated into Dutch by TB.
(0.03 MB DOC)

Alternative Language Abstract S4 Abstract translated into Portuguese by Dr. Jose Sousa-Figueiredo.
(0.03 MB DOC)

Protocol S1 Transmission model.
(0.37 MB DOC)

Protocol S2 Intervention models.
(0.88 MB DOC)

Protocol S3 Bayesian model fitting and parameter values.
(0.92 MB DOC)

Protocol S4 Seasonal patterns and transmission settings.
(0.49 MB DOC)

Protocol S5 Additional results and sensitivity analyses.
(1.26 MB DOC)

Protocol S6 User-friendly software for model runs.
(0.17 MB DOC)

## Abbreviations

ACT: artemisinin-combination therapy
EIR: entomological inoculation rate
EPI: Expanded Program for Immunization
GMEP: Global Malaria Eradication Program
HBI: human blood index
ibppy: infectious bites per person per year
IRS: indoor residual spraying
LLIN: long-lasting insecticide treated net
MSAT: mass screening and treatment
SP: sulphadoxine-pyrimethamine
