# Supplementary material for: Reducing Plasmodium falciparum Malaria Transmission in Africa: A Model-Based Evaluation of Intervention Strategies
Source: PLoS Med. 2010 Aug 10;7(8):e1000324. doi: 10.1371/journal.pmed.1000324 (PMC2919425; doi:10.1371/journal.pmed.1000324)
Supplement: Alternative Language Abstract S1 — Abstract translated into French by Emilie Pothin. (0.03 MB DOC) [file pmed.1000324.s001.doc]

**Résumé**

**Contexte**: Au cours de la derrière décennie, les interventions de contrôle du paludisme se sont largement répandues en Afrique. Cependant, l’impact potentiel de ces mesures sur la transmission doit être quantifié.

**Méthodes et résultats :** Nous avons développé un modèle de simulation individu-centré pour la transmission de *Plasmodium falciparum* en Afrique, prenant en compte les 3 espèces de vecteur principales (*Anophèles gambiae s.s.*, *An. arabiensis* et *An. funestus*). Les valeurs des paramètres ont été obtenues en ajustant le modèle aux données de prévalence issues de 34 études de transmission dans différentes régions d’Afrique. Nous avons incorporé les effets du changement de traitement vers un traitement combiné avec l’artemisinine (TCA) ainsi que l’utilisation croissante depuis 2000 de moustiquaires imprégnées d’insecticide longue durée (MLD). Nous avons ensuite exploré l’impact de l’utilisation continue des MLD, de la pulvérisation d’insecticide à l’intérieur des foyers, du dépistage et traitement de masse, du futur vaccin RTS,S/AS01 sur la transmission du paludisme dans 6 régions caractéristiques ayant des intensités de transmission variables (représenté par le taux annuel d’inoculation entomologique, TIE : 1 faible, 3 modérée et 2 forte), des espèces vectrices et des saisonnalités différentes. Pour chacune de ces régions, nous avons considéré un objectif réaliste de 80% de couverture par les interventions. Dans les régions de faible transmission (TIE env. 3 piqûres infectantes/homme/an), l’utilisation des MLD pourrait ramener la transmission a des niveaux très bas (prévalence parasitaire <1% dans toutes les classes d’âge) sous réserve qu’elle soit fréquente et continue. Dans 2 des régions à transmission modérée (TIE env. 43, 81 piqûres infectante/homme/an), l’ajout de pulvérisations de DDT couplées au dépistage et traitement de masse ramènera la prévalence parasitaire a moins de 1%. Cependant, dans la troisième région à transmission modérée (TIE=46), où *An. Arabiensis* est prédominant, ces interventions ne permettraient pas de ramener la prévalence sous un tel seuil. Dans les 2 régions à forte transmission (TIE env. 586, 675 piqûres infectantes/homme/an), il serait nécessaire d’augmenter le taux de couverture à des niveaux irréalistes (>90%) ou de mettre en place de nouvelles mesures de contrôle et/ou d’obtenir de considérables améliorations sociales ; cependant, une réduction de la prévalence non négligeable pourrait être obtenue grâce aux moyens actuels et des taux de couverture réalistes.

**Conclusions :** Les mesures de contrôle actuellement disponibles peuvent réduire drastiquement la transmission de *P. falciparum* ainsi que le poids de cette maladie en Afrique. La réduction de la prévalence parasitaire au dessous du seuil de 1% est possible dans des régions de transmission faible à modérée, où le vecteur est essentiellement endophile, à condition que des programmes d’interventions complets et viables soient déployés. Dans les régions à forte transmission, ainsi que les régions où le vecteur est essentiellement exophile, des méthodes additionnelles vont être nécessaire pour viser la prévention des piqûres en extérieur (non protégées par les moustiquaires), les moustiques d’extérieur (non concernés par la pulvérisation en intérieur) ainsi que les moustiques zoophiles.
