# Supplementary material for: Reducing Plasmodium falciparum Malaria Transmission in Africa: A Model-Based Evaluation of Intervention Strategies
Source: PLoS Med. 2010 Aug 10;7(8):e1000324. doi: 10.1371/journal.pmed.1000324 (PMC2919425; doi:10.1371/journal.pmed.1000324)
Supplement: Alternative Language Abstract S2 — Abstract translated into Spanish by MGB. (0.03 MB DOC) [file pmed.1000324.s002.doc]

**Resumen**

**Antecedentes:** Durante la última década se ha implementado en África un escalamiento substancial de la cobertura de intervenciones antimaláricas, pero todavía no se ha determinado el impacto general sobre la transmisión de la malaria que puede lograrse utilizando las herramientas de control disponibles hasta el momento.

**Métodos y Hallazgos:** Para tal fin, hemos desarrollado un modelo de simulación individual que incorpora las tres especies vectoras consideradas como las más importantes en la transmisión de *Plasmodium falciparum* en África (a saber, *Anopheles gambiae* s.s., *An. arabiensis* y *An. funestus*). Los parámetros utilizados fueron obtenidos ajustando el modelo a datos de prevalencia parasitaria provenientes de 34 localidades con transmisión malárica a lo largo de África. Incorporamos el efecto del cambio en los esquemas de tratamiento hacia el empleo de combinaciones terapéuticas con derivados de la artemisinina (CTDA), y el incremento en la cobertura de los mosquiteros impregnados con insecticida de larga duración (MILD) que se han producido a partir del año 2000. Procedemos a explorar el impacto sobre la transmisión de una expansión continua de MILD, rondas adicionales de rociamientos intra-domiciliarios con insecticidas de acción residual (RII), rastreo masivo y tratamiento (RMT), y una futura vacuna RTS,S/AS01, en seis localidades representativas de las variaciones en la intensidad de transmisión (medida por la tasa de inoculación entomológica (TIE): una con baja, tres con moderada, y dos con elevada transmisión), combinaciones de especies vectoras, y patrones de estacionalidad. En todas las localidades consideramos como meta realista el logro de un 80% en la cobertura de las intervenciones. En la localidad con baja transmisión (TIE aproximada de 3 picadas infectivas por persona por año), MILD tiene el potencial de reducir la transmisión de la malaria a niveles por debajo del 1% en la prevalencia parasitaria en todos los grupos de edad, siempre que los niveles de uso de dichos mosquiteros sean elevados y sostenidos. En dos de las localidades de transmisión moderada (TIE aproximada de 43 y 81 respectivamente), se requerirían rondas adicionales de RII con DDT acoplados con RMT para reducir la prevalencia parasitaria por debajo del umbral del 1%. Sin embargo, en la tercera localidad de transmisión intermedia (TIE=46), pero con una preponderancia de *An. arabiensi*s, dichas intervenciones resultarían insuficientes para alcanzar el mencionado umbral. En las dos localidades de transmisión intensa (TIE aproximadamente igual a 586 y 675 respectivamente), habría que alcanzar niveles de cobertura superiores al 90% (que serían poco realistas), o se necesitarían nuevas herramientas de control, o mejoras substanciales en los niveles socio-económicos de las poblaciones afectadas. Esto no descarta la posibilidad de que los niveles de prevalencia parasitaria puedan reducirse considerablemente mediante la utilización de las medidas de control actualmente disponibles con coberturas más realistas.

**Conclusiones:** Nuestro modelo indica que intervenciones antimaláricas basadas en las medidas de control existentes pueden reducir significativamente la transmisión de *P. falciparum* asociada con la carga de morbilidad en África. En aquellas áreas que originalmente tienen una transmisión baja a intermedia, y donde las especies vectoras son principalmente endofílicas, sería posible reducir la prevalencia parasitaria por debajo del umbral del 1% siempre y cuando los programas de control logren una expansión comprehensiva y sostenible de las intervenciones. En aquellas áreas en las cuales los vectores son mayormente exofílicos, o la transmisión es elevada, se necesitan medidas adicionales que reduzcan la transmisión peri-domiciliaria por mosquitos que pican y descansan fuera de las casas, o que son parcialmente zoofágicos.
