# Supplementary material for: Reducing Plasmodium falciparum Malaria Transmission in Africa: A Model-Based Evaluation of Intervention Strategies
Source: PLoS Med. 2010 Aug 10;7(8):e1000324. doi: 10.1371/journal.pmed.1000324 (PMC2919425; doi:10.1371/journal.pmed.1000324)
Supplement: Alternative Language Abstract S3 — Abstract translated into Dutch by TB. (0.03 MB DOC) [file pmed.1000324.s003.doc]

**Samenvatting (Nederlands)**

**Achtergrond:** In het afgelopen decennium is de dekkinggraad van malaria interventies toegenomen in Afrika. Het blijft echter onduidelijk welke reductie in transmissie intensititeit haalbaar is met de momenteel beschikbare middelen.

**Methoden en resultaten:** We hebben een microsimulatie model ontwikkeld voor de transmissie van *Plasmodium falciparum* in de Afrikaanse context met de 3 belangrijkste vector species (*Anopheles gambiae s.s., An. arabiensis* en *An. funestus*), waarbij parameters bepaald werden aan de hand van kalibratie op prevalentiedata uit 34 transmissiegebieden in Afrika. We includeerden het effect van de overstap op artemisine-combinatietherapie (ACT) en toenemende dekking met langwerkende insecticide behandelde netten (LLIN) sinds het jaar 2000. Vervolgens onderzochten we de gevolgen op transmissie van de voortgaande implementatie van LLINs, additionele rondes met binnenshuis sproeien met insecticiden (IRS), grootschallige screening en behandeling (MSAT) en een toekomstig RTS,S/AS01 vaccin in zes gebieden die representatief zijn voor gebieden met variërende transmissie intensiteit (samengevat door een jaarlijkse entomologische inoculatie graad , EIR: 1 situatie met lage, 3 met matige en 2 met hoge EIRs) , vector species combinaties en seizoenspatronen. In alle situaties namen we 80% aan als realistisch doel voor de dekkingsgraad. In de laag endemische setting (EIR ongeveer 3 infectieuze beten per person per jaar (ibppj), kunnen LLINs malaria transmissie verlagen tot lage intensiteit (<1% parasite prevalentie in alle leeftijdsgroepen) zolang gebruikersniveau’s hoog zijn en kunnen worden gehouden. In twee van de gebieden met matige transmissie-intensiteit (EIR ongeveer 43, 81 ibppj) zouden additionele rondes van IRS met DDT in combinatie met MSAT de parasite prevalentie onder de 1% drempel kunnen krijgen. Echter, in een derde gebied (EIR=46) met voornamelijk *An. arabiensis*, zijn deze interventies onvoldoende om deze drempel te bereiken. In beide hoog endemische gebieden (EIR ongeveer 586, 675 ibppj) zijn ofwel onrealistisch hoge dekkingsgraden nodig (>90%) ofwel nieuwe middelen en/of aanzienlijke sociale verbeteringen, hoewel een aanzienlijk reductie in prevalentie verwezenlijkt kan worden met de momenteel beschikbare middelen en een realistische dekkingsgraad.

**Conclusies:**

Interventies met momenteel beschikbare middelen kunnen aanzienlijke reducties in *P. falciparum* malaria transmissie en de daaraan gekoppelde ziektelast bewerkstelligen in Afrika. Reductie tot de 1% drempel van parasiet prevalentie is mogelijk in laag en matig endemische gebieden als vectoren voornamelijk endofiel zijn, als een uitgebreid interventie programma wordt geïmplementeerd en volgehouden. In hoog endemische situaties en situaties waarbij vectoren vooral exofiel zijn, zijn additionele middelen nodig die zich richten op buitenshuis bijtende en rustende muggen en muggen die deels op dieren voeden.
