# Supplementary material for: Reducing Plasmodium falciparum Malaria Transmission in Africa: A Model-Based Evaluation of Intervention Strategies
Source: PLoS Med. 2010 Aug 10;7(8):e1000324. doi: 10.1371/journal.pmed.1000324 (PMC2919425; doi:10.1371/journal.pmed.1000324)
Supplement: Alternative Language Abstract S4 — Abstract translated into Portuguese by Dr. Jose Sousa-Figueiredo. (0.03 MB DOC) [file pmed.1000324.s004.doc]

**Sumário** 

**Antecedentes**: Durante a última década, a cobertura de intervenções anti-malária em África sofreram um aumento significativo. No entanto, ainda está por ser determinado o impacto destas intervenções na transmissão da malária, que têm por base ferramentas de controle actualmente disponíveis.

**Métodos e Resultados**: Desenvolvemos um modelo de simulação que incorpora três espécies de vectores na transmissão de *Plasmodium falciparum* em África (*Anopheles gambiae ss*, *A. arabiensis* e *A. funestus*). Os parâmetros utilizados foram obtidos pelo ajuste do modelo de acordo com dados de prevalência de malária em 34 áreas em África. Incorporamos o efeito das alterações nos padrões de tratamento em relação ao uso de combinações terapêuticas com derivados de artemisinina, e aumento da cobertura de mosquiteiros impregnados de longa duração (MILD) a partir de 2000. Exploramos o impacto na transmissão de malária após uma expansão contínua de MILDs, rodadas de pulverização residual intra-domiciliar (PRID) com insecticidas, monitoramento e tratamento massivo (MTM) e uma futura vacina RTS, S/AS01 em seis localidades representativas das variações na intensidade de transmissão (medido pela taxa de inoculação entomológica (TIE): uma localidade com uma TIE reduzida, três com moderada e duas com elevada), das combinações de espécies de vectores, e dos padrões de sazonalidade. Em todas as localidades nós consideramos como meta realista alcançar uma cobertura de 80% das intervenções. Na localidade de transmissão reduzida (TIE = 3 picadas infectantes por pessoa por ano), MILDs têm o potencial para reduzir a transmissão da malária para níveis abaixo de 1% de prevalência em todas as faixas etárias, desde que a utilização seja contínua e elevada. Em duas das localidades de transmissão moderada (TIE = 43 e 81), rodadas adicionais de PRID, juntamente com MTM, seriam necessárias para reduzir a prevalência de parasitas para baixo de 1%. No entanto, na terceira localidade de transmissão moderada (TIE = 46), com uma preponderância de *A. arabiensis*, essas intervenções não seriam suficientes para atingir o limiar supra mencionado. Nas duas localidades de transmissão elevada (TIE = a 586 e 675) seria necessário atingir níveis de cobertura acima de 90%, o que seria irrealista, a menos que se implantasse novas ferramentas de gestão ou se alcançasse melhorias substanciais nos níveis socio-económicos das populações afectadas. Isto não exclui a possibilidade dos níveis de prevalência de parasitas poderem ser reduzidos consideravelmente usando as medidas de controle atualmente disponíveis, mas com uma cobertura mais realista.

**Conclusões**: O nosso modelo sugere que as intervenções anti-malária com base em medidas de controle existentes podem reduzir significativamente a transmissão de *P. falciparum* e, consequentemente, reduzir a morbilidade e mortalidade causadas pela malária, em África. Em áreas de transmissão reduzida/moderada e onde as espécies de vectores são maioritariamente endófilas, será possível reduzir a prevalência de parasitas abaixo do limiar de 1% com uma expansão sustentável de intervenções. Em áreas onde os vetores são maioritariamante exofílicos, ou a transmissão é elevada, serão necessárias medidas adicionais para reduzir a transmissão peri-domiciliar por mosquitos que picam e descansam fora da casa, ou são parcialmente zoofágicos.
