# Supplementary material for: Reducing Plasmodium falciparum Malaria Transmission in Africa: A Model-Based Evaluation of Intervention Strategies
Source: PLoS Med. 2010 Aug 10;7(8):e1000324. doi: 10.1371/journal.pmed.1000324 (PMC2919425; doi:10.1371/journal.pmed.1000324)
Supplement: Protocol S1 — Transmission model. (0.37 MB DOC) [file pmed.1000324.s005.doc]

**Reducing *Plasmodium falciparum* malaria transmission in Africa: a model-based evaluation of intervention strategies**

Jamie T Griffin^1^, T. Deirdre Hollingsworth^1^, Lucy C Okell^1^, Thomas S Churcher^1^, Michael White^1^, Wes Hinsley^1^, Teun Bousema^2^, Chris J Drakeley^2^, Neil M Ferguson^1^, María-Gloria Basáñez^1^, Azra C Ghani^1^.

1. *MRC Centre for Outbreak Analysis & Modelling, Department of Infectious Disease Epidemiology, Imperial College London*
2. *Department of Infectious Diseases, London School of Hygiene & Tropical Medicine*

# PROTOCOL S1

**TRANSMISSION MODEL**

In the paper we use a simulation model in which individuals are modeled alongside a stochastic population of the three main vector species. However, the processes parallel a compartmental deterministic model used for model validation. We therefore begin by setting out the mathematical syntax of the basic transmission model in its compartmental formulation after which we describe its individual-based formulation. For the interventions, we again set out deterministic formulations of these models as extensions of previous models before describing their implementation within an individual-based framework.

## Compartmental Model

### Human Model

At each point in time people can be in one of six infectious states – susceptible (S), treated clinical disease (T), untreated clinical disease (D), asymptomatic patent infection (A), sub-patent infection (U) and protected by a period of prophylaxis from prior treatment (P). People move between these states as shown in Figure S1.1, with rates/probabilities as marked on the arrows and described in the main text. Latent liver stage infection is not modeled explicitly but rather captured as a delay in the force of infection of duration $d_{E}$. This allows the infection process to be identical for susceptible individuals and for those re-infected via superinfection.

**Figure S1.1. Flow diagram for the human infection states**

The model partial differential equations for the human dynamics are as follows, with *t* representing time and *a* age,

The probability of infection *b* (included in the force of infection Λ), the probability of clinical disease and the recovery rate from asymptomatic infection $r_{A}$ all vary with immunity as described in Section 1.1.3. The force of infection $\Lambda$ also varies by age due to increasing biting rates as described in Section 1.1.2.

### Heterogeneity in biting rates

The rate at which people are bitten is assumed to vary with age, due to increasing body size [1,2]. We also assume that each person has a relative biting rate ζ which persists over their lifetime, with ζ having a log-normal distribution between people with a mean of 1,

$\log(\zeta)\sim N(-\sigma^{2}/2, \sigma^{2})$.

Hence the force of infection at age *a* and time *t* is given by

$\Lambda(a,t)=\varepsilon_{0}(t)\zeta b\psi(a)$,

where $\psi$ is the relative biting rate at age *a*,

$\psi(a)=\left(1-\rho\exp(-a/a_{0})\right)$.

$\varepsilon_{0}$ is the EIR as measured for adults at time *t*, 1-*ρ* is the relative biting rate at birth compared to adults, *a*_0_ determines the time-scale of the increase in biting rate with age and *b* is the probability of infection if bitten by an infectious mosquito.

### Human Immunity functions

We consider three transition points at which immunity may act: a reduction in the probability that infection is established following an infectious challenge (pre-erythrocytic immunity, *I_B_*), a reduction in the probability of clinical disease upon infection (clinical immunity, *I_C_*), and a shorter duration of patent parasitemia (blood-stage immunity, *I_A_*). For model fitting purposes we considered these as separate functional forms that could depend on either age alone (i.e. a pure physiological process), exposure alone (as explained below) or a combination of both age and exposure.

Immunity to infection, $I_{B}(a,t)$ in a population exposed to an EIR $\varepsilon(a,t)$ is a function of both age and time and is given by the partial differential equation,

$\frac{\partial I_{B}}{\partial t}+\frac{\partial I_{B}}{\partial a}=h_{B}(\varepsilon(a,t)) -I_{B}/d_{B}, \begin{matrix} & \end{matrix}I_{B}(0,t)=0$,

where $h_{B}(.)$is the assumed immunity acquisition profile described below, and *d_B_* is the mean duration of immunity to infection. The probability of infection by age is then given by a Hill function,

,

where , $b_{MIN}$, $I_{B0}$ and $\kappa_{B}$ are parameters estimated during model fitting.

Immunity to clinical disease, *I_C_* comprises that acquired by exposure to infection (*I_CA_*) and that maternally acquired (*I_CM_*). $I_{CA}(a,t)$ in a population exposed to a force of infection $\Lambda$is a function of both age and time and is given by the partial differential equation,

$\frac{\partial I_{CA}}{\partial t}+\frac{\partial I_{CA}}{\partial a}=h_{C}(\Lambda) -I_{CA}/d_{C}, \begin{matrix} \begin{matrix} & \end{matrix} & \end{matrix}I_{CA}(0,t)=0$,

where again $h_{C}(.)$is the assumed immunity acquisition profile described below, and *d_C_* is the mean duration of clinical immunity. Maternally-acquired immunity $I_{CM}(a,t)$is assumed at birth to be a proportion (*P_CM_*) of the level of immunity present in a 15- to 30-year old woman (taken to be a 20-year-old here), *I_C_* (20, *t*), living in the same location and which decays at a constant rate (1/*d_M_*),

$\frac{\partial I_{CM}}{\partial t}+\frac{\partial I_{CM}}{\partial a}= -I_{CM}/d_{M}, I_{CM}(t,0)=P_{CM}I_{C}(20,t)$.

Thus the total clinical immunity by age and time is given by $I_{C}=I_{CA}+I_{CM}$. The probability of acquiring clinical disease upon infection by age is then given by a Hill function,

,

where $I_{C0}$ and $\kappa_{C}$ are parameters estimated during model fitting.

For both anti-infection and clinical immunity we considered four different choices for $h(X)$in the fitting procedure outlined in Protocol S3, where X is either the EIR or force of infection:

1. No immunity: I=0
2. A purely physiological process where immunity increases with age only: $h(X)=1$
3. Immunity increases linearly with exposure: $h(X)=X$
4. Immunity increases with exposure but with a period $\gamma$ after each exposure in which immunity is not boosted, resulting in an increase at average rate $h(X)=\frac{X}{\gamma X+1}$

Finally blood-stage immunity that reduces parasite density and hence shortens the period of patent infection, $I_{A}$, is given by the partial differential equation,

$\frac{\partial I_{A}}{\partial t}+\frac{\partial I_{A}}{\partial a}=h_{A}(\Lambda)-I_{A}/d_{A}, I_{A}(t,0)=0$.

We assume here that anti-parasite immunity is only physiological (and thus that $h_{A}(\Lambda)=1$) and 1/*d_A_* is its constant rate of decay. Then the recovery rate from patent infection as a function of age is given by,

##### $r_{A}(a)=r_{A0}\left(1+(w_{A}-1)\frac{(I_{A}/I_{A0})^{\kappa_{A}}}{1+(I_{A}/I_{A0})^{\kappa_{A}}}\right)$,

where$r_{A0}$ is the rate for non-immune individuals (i.e. infants).

### Mosquito model

The three main *Anopheles* species that transmit *Plasmodium falciparum* in Africa are included separately in the model. They are denoted by the superscript $v$, be it *An. gambiae s.s.* ($v$=1), *An. arabiensis* ($v$=2) or *An. funestus* ($v$=3). The characteristics of each vector species are outlined in Protocol S3. Each vector can be in one of three states, susceptible ($S_{M}^{v}$), latently infected ($E_{M}^{v}$) and infectious ($I_{M}^{v}$). The dynamics of infection in the mosquito is given by the set of differential equations,

where is the probability that a mosquito of species $v$ survives the period from acquiring infection until sporozoites appear in the salivary glands ($\tau_{M}$, extrinsic incubation period or EIP), $\mu^{v}$ is the death rate, $\Lambda_{M}^{v}$ is the force of infection acting on mosquito of species $v$ and $\beta^{v}(t)$ is the species-specific time-varying emergence rate which is fitted to data from the different transmission sites (see Protocol S4). It is assumed that the population dynamics of each species is not affected by the presence or absence of the other species (i.e. there is no interspecies competition) and that mosquito mortality does not depend on infection status.

The human adult EIR due to each species is given by $\varepsilon^{v}=\frac{\alpha^{v}I_{M}^{v}}{\omega}$ where $\alpha^{v}$ is the rate at which a mosquito of species $v$ takes a human blood meal and $\omega$ is the normalizing constant for the biting rate over ages, with a population age distribution of $\eta(a)$,

$\omega=\int_{0}^{\infty} \eta(a)\psi(a) da$.

The overall EIR is therefore simply$\varepsilon_{0}=\varepsilon^{1}+\varepsilon^{2}+\varepsilon^{3}$. The force of infection acting on mosquitoes is the sum of the contribution to mosquito infection from the different human infectious states,

$\Lambda_{M}^{v}(t)=\frac{\alpha^{v}}{\omega}\int_{\zeta} \int_{a} \zeta\psi(a)\left(c_{D}D(\zeta,a,t-t_{l})+c_{T}T(\zeta,a,t-t_{l})+c_{A}A(\zeta,a,t-t_{l})+c_{U}U(\zeta,a,t-t_{l})\right) da d\zeta$,

where $c_{D}$, $c_{T}$, $c_{A}$ and $c_{U}$ are the onward infectivity to mosquitoes of these different states and $t_{l}$ is the time-lag between parasitemia with asexual parasite stages and gametocytemia (infectivity to mosquitoes) to account for the lag in gametocyte development characteristic of *P. falciparum*.

## Simulation Model

The simulation follows the same processes as the population-level model but humans are explicitly modeled individually. The vector dynamics are modeled as a stochastic version of the compartmental model. At initialization, the age structure of the population is drawn from an exponential distribution parameterized with data from Tanzania [3]. Age-specific infection states and immunity status are also drawn from the equilibrium solution for the compartmental model. Each individual in the population is assigned to a heterogeneity category at birth or model initialization based on the distribution in Section 1.1.2 which they keep for their lifetime and a scheduled time of death.

An individual’s infection status is tracked over time and follows the diagram shown in Figure S1.1, and their immunity status is also tracked and increased or decreased when necessary in accordance with the equations in Section 1.1.3.

For each infectious state, we calculate each person’s contribution to the force of infection on mosquitoes $\Lambda_{M}^{v}$. Let *i* index people, and $\zeta_{i}$ be the relative rate at which person *i* is bitten. The proportion of the whole population’s bites that person *i* receives is

$$\pi_{i}=\frac{\zeta_{i}\psi(a_{i})}{\sum_{i} \zeta_{i}\psi(a_{i})}$$

The mean infectivity to mosquitoes of the whole infected and uninfected population, weighted by how frequently different people are bitten, is then $C=\sum_{i} \pi_{i}c_{i}$, where *c_i_* is the infectivity of the infectious state that person *i* is in.

Allowing a lag $t_{l}$ between human asexual parasite stages and infectivity to mosquitoes to account for gametocyte development, the force of infection on mosquitoes is then given by,

$\Lambda_{M}^{v}(t)=\alpha^{v}C(t-t_{l})$,

where $\alpha^{v}$ is the species-specific rate at which a mosquito takes a human blood meal.

The three mosquito populations are modeled using the stochastic equivalent of the set of deterministic equations given in Section 1.1.4. In the individual-based model the EIR for person *i* is given by$\pi_{i}\sum_{\text{v=1}}^{3} \alpha^{v}I_{M}^{v}(t)$.

## References
