# Supplementary material for: Reducing Plasmodium falciparum Malaria Transmission in Africa: A Model-Based Evaluation of Intervention Strategies
Source: PLoS Med. 2010 Aug 10;7(8):e1000324. doi: 10.1371/journal.pmed.1000324 (PMC2919425; doi:10.1371/journal.pmed.1000324)
Supplement: Protocol S2 — Intervention models. (0.88 MB DOC) [file pmed.1000324.s006.doc]

**Reducing *Plasmodium falciparum* malaria transmission in Africa: a model-based evaluation of intervention strategies**

Jamie T Griffin^1^, T. Deirdre Hollingsworth^1^, Lucy C Okell^1^, Thomas S Churcher^1^, Michael White^1^, Wes Hinsley^1^, Teun Bousema^2^, Chris J Drakeley^2^, Neil M Ferguson^1^, María-Gloria Basáñez^1^, Azra C Ghani^1^.

1. *MRC Centre for Outbreak Analysis & Modelling, Department of Infectious Disease Epidemiology, Imperial College London*
2. *Department of Infectious Diseases, London School of Hygiene & Tropical Medicine*

# PROTOCOL S2

**INTERVENTION MODELS**

# Intervention Models

## Long-lasting insecticide treated nets (LLINs) and Indoor Residual Spraying (IRS)

LLINs and IRS have four main effects on the transmission cycle:

1. they increase the overall mosquito death rate;
2. they lengthen the feeding or gonotrophic cycle;
3. they change the proportion of bites taken on protected and unprotected people;
4. they change the proportion of bites taken on humans relative to animals (the Human Blood Index).

Highly effective LLIN and IRS campaigns that substantially reduce the size of the mosquito population may reduce vector density further by reducing mosquito emergence. However, these additional benefits of effective vector control programs have not been disentangled from the direct reductions in vector density caused by mosquito mortality and can be compensated for if there is density-dependent mosquito larvae survival or other fitness components in breeding sites. The population dynamics of the mosquito are also difficult to estimate in the field and are likely to depend on multiple non-linear processes which may reduce the impact that vector control will have on mosquito emergence. Accurately quantifying these downstream benefits of LLIN and IRS is therefore beyond the scope of this paper, so our estimates of the benefits of such interventions will tend to be conservative in areas where they are highly effective.

To model the impact that LLIN and IRS campaigns will have on the vector population we extend the approach outlined by Le Menach et al. [1] to incorporate human age structure, heterogeneous biting rates, more realistic population coverage and the additional effect of IRS. By calculating the effect over individuals rather than in compartments, we are able to represent more accurately the coverage patterns seen in real populations (e.g. nets may not always be distributed to the same person in multiple distribution campaigns). This allows us to investigate the impact of more realistic age-targeted approaches including distribution to newborn infants and their parents whilst capturing the decay in net efficacy, physical condition and adherence to usage.

The probability of a blood-seeking mosquito successfully feeding will depend on the behavior of the mosquito (which may vary between species) and the anti-vectorial defenses employed by the human host population. A graphical representation of different possible outcomes of a mosquito feeding attempt in the presence of LLINs is given in Figure S2.1. Epidemiologically, the model assumes that there are 5 different outcomes of a mosquito attempting to feed: 1) it bites a non-human host; 2) it is killed by the LLIN before it bites; 3) it is killed by IRS after it bites; 4) it successfully feeds and survives that feeding attempt; 5) it is repelled without feeding, either through the actions of LLIN or IRS. Repelled mosquitoes then go on to find alternative blood meal sources (a process referred to as repeating). It is assumed that all cattle are kept outside of the house and therefore all mosquitoes that enter the house attempt to bite humans.

Assume that person *i* is protected by a given LLIN/IRS efficacy. We define the probability of a mosquito of species $v$biting host *i* during a single attempt to be $y_{i}^{v}$; the probability that a mosquito bites a host and survives the feeding attempt to be $w_{i}^{v}$, and the probability of it being repelled without feeding to be $z_{i}^{v}$. These probabilities exclude natural vector mortality, so that for someone with no protection, $y_{i}^{v}=w_{i}^{v}=1$ and $z_{i}^{v}=0$. It should be noted, however, that not all mosquitoes successfully feed when they enter a house so that estimates of$y_{i}^{v}$,$w_{i}^{v}$ and $z_{i}^{v}$must take into account the repeating behavior observed prior to the introduction of insecticides.

**Figure S2.1. Flow chart of mosquito life cycle based on the diagram from Le Menach *et al.* [1]. Note the addition of an extra class of human for those people not sleeping under bed nets.**


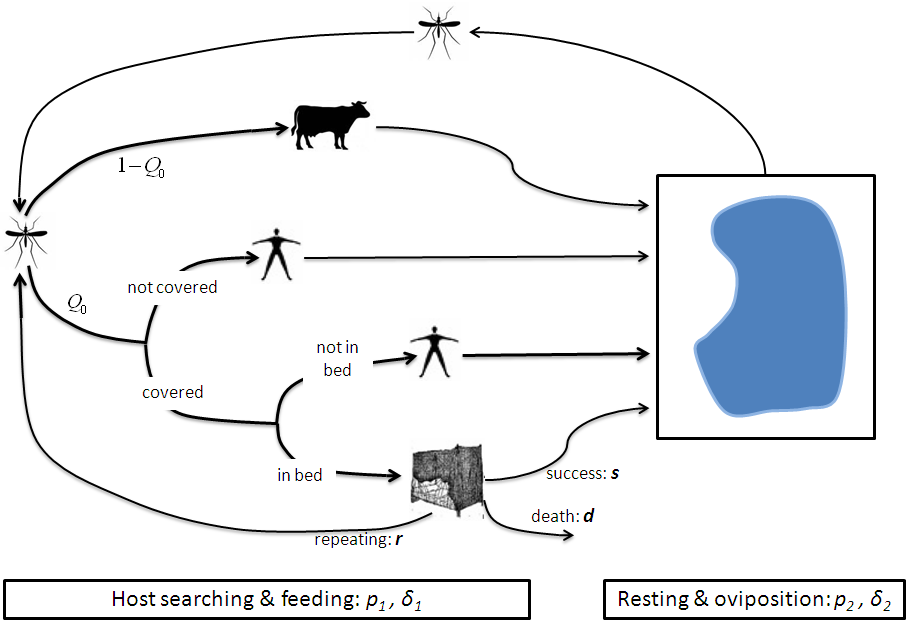


Following the approach outlined by Le Menach et al. [1] during a single feeding attempt (which may be on animals or humans), a mosquito of species $v$ will successfully feed with probability $W^{v}$ given by,

$W^{v}=1-Q_{0}^{v}+Q_{0}^{v}\sum_{i} \pi_{i}w_{i}^{v}$,

and be repelled without feeding with probability $Z^{v}$ given by

$Z^{v}=Q_{0}^{v}\sum_{i} \pi_{i}z_{i}^{v}$,

where in both equations $Q_{0}^{v}$is the proportion of bites taken on humans by species $v$ in the absence of any intervention and $\pi_{i}$ is the proportion of bites on humans that person *i* receives, also in the absence of any intervention.

**Table S2.1: Explanation of key mosquito repellency, feeding success and death terms.**

| **Expression** | **Description** |
| --- | --- |
| $r_{N}, r_{S}$ | probability of repeating upon encounter with net/IRS |
| $d_{N}, d_{S}$† | probability of dying upon encounter with net/IRS |
| $s_{N}, s_{S}$† | probability of successful feeding upon encounter with net/IRS |
| $z_{i}$ | probability of mosquito being repelled from host *i* without feeding during a single attempt |
| $y_{i}$ | probability of mosquito feeding on host *i* during a single attempt |
| $w_{i}$ | probability of mosquito feeding and surviving on host *i* during a single attempt |
| $Z$ | average probability of mosquito repeating during a single attempt |
| $W$ | average probability of successfully feeding on a human during a single attempt |

† With IRS, but not with LLINs, s and d are conditional on not being repelled.

The length of time spent looking for a blood meal and resting between feeds are $\delta_{1}^{v}$ and $\delta_{2}^{v}$ respectively. The mosquito feeding rate $f_{R}^{v}$ is given by $f_{R}^{v}=\frac{1}{\delta_{1}^{v}+ \delta_{2}^{v}}$. Parameter $\delta_{2}^{v}$ is assumed to be unaffected by the interventions, whilst $\delta_{1}^{v}$ is increased to $\delta_{1}^{v}=\frac{\delta^{v}_{10}}{1-Z^{v}}$ where $\delta^{v}_{10}$ is the value with no interventions.

The probabilities of surviving the periods of feeding and resting are $p_{1}^{v}$ and $p_{2}^{v}$*.* With no interventions,

$p_{10}^{v}=\exp(-\mu_{0}^{v}\delta_{10}), p_{2}^{v}=\exp(-\mu_{0}^{v}\delta_{2})$,

where $\mu_{0}^{v}$is the natural death rate of mosquito species $v$. With interventions $p_{2}^{v}$is unchanged and $p_{1}^{v}= \frac{p_{10}^{v}w^{v}}{1-Z^{v}p_{10}^{v}}$.

The probability of surviving one feeding cycle is $p_{1}^{v}p_{2}^{v}$. Hence the mosquito death rate $\mu^{v}$can be found as,

$\begin{matrix} p_{1}^{v}p_{2}^{v}=\exp(-\mu^{v}/f_{R}^{v}) \\ \mu^{v}=-f_{R}^{v}\log(p_{1}^{v}p_{2}^{v}) \end{matrix}$.

The probability of surviving the extrinsic incubation period, $P_{M}^{v}$, therefore also changes as* *$\mu^{v}$ changes.

The probability that a feeding cycle ends with a successful bite on person *i* ,$q_{i}^{v}$, is,

$\begin{matrix} q_{i}^{v}=p_{10}^{v}\left(Q_{0}^{v}\pi_{i}w_{i}^{v}+Z^{v}q_{i}^{v}\right) \\ q_{i}^{v}=\frac{p_{10}^{v}Q_{0}^{v}\pi_{i}w_{i}^{v}}{1-Z^{v}p_{10}^{v}} \end{matrix}$.

The probability that a feeding cycle ends with a bite on an animal is,

$\begin{matrix} q_{A}^{v}=p_{10}^{v}\left(1-Q_{0}^{v}+Z^{v}q_{A}^{v}\right) \\ q_{A}^{v}=\frac{p_{10}^{v}(1-Q_{0}^{v})}{1-Z^{v}p_{10}^{v}} \end{matrix}$.

Hence the proportion of successful bites which are on humans is,

$$\begin{matrix} Q^{v}=1-\frac{q_{A}^{v}}{q_{A}^{v}+\sum_{i} q_{i}^{v}}=1-\frac{(1-Q_{0}^{v})}{(1-Q_{0}^{v})+Q_{0}^{v}\sum_{i} \pi_{i}w_{i}^{v}} \\ =1-\frac{(1-Q_{0}^{v})}{W^{v}} \end{matrix}$$

and the biting rate on humans is,

$\alpha^{v}=Q^{v}f_{R}^{v}$.

The rate at which person *i* is bitten by a single mosquito of species $v$ is,

$\lambda_{i}^{v}=\frac{\alpha^{v}\pi_{i}w_{i}^{v}}{\sum_{i} \pi_{i}w_{i}^{v}}$,

and the force of infection on mosquitoes is,

$\Lambda_{M}^{v}=\sum_{i} c_{i}\lambda_{i}^{v}$.

When IRS is used, some mosquitoes may bite a person before dying by picking up a lethal insecticide dose when resting on the walls of the house. So for calculating the force of infection on humans, the biting rate on each person needs to be inflated by a factor $\frac{y_{i}^{v}}{w_{i}^{v}}$giving an effective biting rate,

$\lambda_{i}^{v}*=\frac{\alpha^{v}\pi_{i}y_{i}^{v}}{\sum_{i} \pi_{i}w_{i}^{v}}$.

The EIR experienced by person *i* due to this mosquito species is $\lambda_{i}^{v}*I_{M}^{v}$ and the total EIR they experience is the sum of this over the vector species present.

#### Expressions for $w_{i}^{v}$, $y_{i}^{v}$ and $z_{i}^{v}$

The degree of protection afforded by LLIN and IRS will depend on the proportion of bites humans receive whilst protected by the intervention [2,3]. This will depend on host movement / sleeping patterns, the biting behavior of the mosquito vector and the efficacy of the intervention.

Let the rate at which a person who is indoors at hour *t* is bitten be $\lambda_{I}(t)$ , and the corresponding figure for someone outdoors be $\lambda_{O}(t)$. Knowing the proportion of human hosts indoors $p_{I}(t)$ or in bed $p_{B}(t)$ at a given time *t* enables us to calculate the proportion of bites taken on humans whilst they are indoors as,

$\Phi_{I}=\frac{\sum_{t} p_{I}(t)\lambda_{I}(t)}{\sum_{t} \left((1-p_{I}(t))\lambda_{O}(t)+p_{I}(t)\lambda_{I}(t)\right)}$,

whereas the proportion of bites taken on the human population whilst they are in bed is,

$\Phi_{B}=\frac{\sum_{t} p_{B}(t)\lambda_{I}(t)}{\sum_{t} \left((1-p_{I}(t))\lambda_{O}(t)+p_{I}(t)\lambda_{I}(t)\right)}$.

Due to the lack of data it is assumed that human movement and sleeping patterns are not dependent on age or relative exposure.

Once a mosquito enters a house to feed, one of three things can happen: it can repeat (*r*), feed successfully (s) or die (d). Let $r_{N},s_{N},d_{N}$ denote repetition, success or death caused by a LLIN and$r_{S},s_{S},d_{S}$ signify repetition, success or death due to IRS. Figure S2.2 shows the order in which the different processes operate when a mosquito attempts to feed on a person protected with both LLIN and IRS. Death from LLINs is assumed to occur before feeding, whereas death from IRS happens after feeding.

**Figure S2.2. Combined model for IRS and LLIN interventions**.


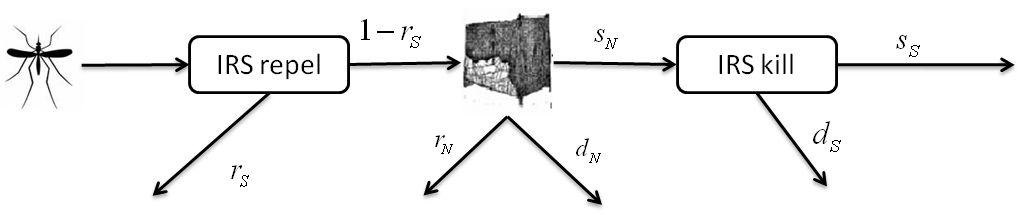


The calculations for the probability of successful feeding, biting and repellency depend on the combination of LLIN/IRS in place in the household where the individual resides, which we assume is an individual-level characteristic. Dropping the superscript $v$, for someone who is unprotected, $y_{i}=w_{i}=1$and $z_{i}=0$, otherwise the expressions are given in Table S2.2. For each outcome, the probability is given by

$$\begin{matrix} \left(1-\Phi_{I}\right)\Pr(\text{outcome with no protection) } \\ + \Phi_{ B}\Pr(\text{outcome with LLIN and IRS protection at the level this person has)} \\ + (\Phi_{I}-\Phi_{B})\Pr(\text{outcome with just IRS protection at the level this person has)} \end{matrix}$$

**Table S2.2**. Probabilities of successful feeding, biting and repulsion for combinations of LLIN/IRS interventions

|  | IRS only | LLINs only | IRS plus LLINs |
| --- | --- | --- | --- |
| Probability of successful feeding (*w_i_*) | $1-\Phi_{I}+\Phi_{I}(1-r_{S})s{}_{S}$ | $1-\Phi_{B}+\Phi_{B}s_{N}$ | $\begin{matrix} 1-\Phi_{I}+\Phi_{ B}(1-r_{S})s_{N}s_{S} \\ +(\Phi_{I}-\Phi_{B})(1-r_{S})s_{S} \end{matrix}$ |
| Probability of biting (*y_i_*) | $1-\Phi_{I}+\Phi_{I}(1-r_{S})$ | $1-\Phi_{B}+\Phi_{B}s_{N}$ | $\begin{matrix} 1-\Phi_{I}+\Phi_{B}(1-r_{S})s_{N} \\ +(\Phi_{I}-\Phi_{B})(1-r_{S}) \end{matrix}$ |
| Probability of repellency (*z_i_*) | $\Phi_{I}r_{S}$ | $\Phi_{B}r{}_{N}$ | $\Phi_{B}(1-r_{S})r_{N}+\Phi_{I}r_{S}$ |

The repellency and mortality effects of IRS start at an optimum value ($r_{S0}$,$d_{S0}$respectively) at the time of spraying and then decrease at a constant rate over time ($\gamma_{\text{S}}$). The effectiveness of IRS depends on the degree of endophily, $\chi$ (the proportion of mosquitoes resting on the wall long enough to be killed by the insecticide), which is known to vary between mosquito species [4]. Hence at time *t* after IRS has been applied,

$$\begin{matrix} r_{S}=r_{S0}\exp(-t\gamma_{S}) \\ d_{S}=\chi d_{S0}\exp(-t\gamma_{S}) \\ s_{S}=1-d_{s} \end{matrix}$$

The repellency of LLIN decreases from a maximum, $r_{N0}$, to a non-zero level$r_{NM}$, reflecting the protection still provided by a net that no longer has any insecticidal effect (and potentially some holes). The killing effect of LLIN decreases from $d_{N0}$ at a constant rate $\gamma_{N}$. So at time *t* after nets were distributed,

$\begin{matrix} r_{N}=(r_{N0}-r_{NM})\exp(-t\gamma_{N})+r_{NM} \\ d_{N}=d_{N0}\exp(-t\gamma_{N}) \\ s_{N}=1-r_{N}-d_{N} \end{matrix}$.

#### Estimates for *r*, *s* and *d*.

The number of mosquitoes entering a house in search of a blood meal can be estimated from experimental hut trials. Different studies report results in different formats though most studies can be summarized (for each vector) as in Table S2.3 (dropping the superscript $v$).

**Table S2.3. The format and notation of experimental studies used to estimate the probability of successful feeding, biting and repellency caused by LLIN (assuming test and control houses were matched prior to the introduction of LLIN)**

|  | Without LLIN | With LLIN |
| --- | --- | --- |
| Number of mosquitoes entering the house | $N_{0}$ | $N_{1}$ |
| % of mosquitoes not feeding | $j_{0}$ | $j_{1}$ |
| % succeeding in feeding | $k_{0}$ | $k_{1}$ |
| % dying | $l_{0}$ | $l_{1}$ |

The presence of a bed net will cause a mosquito to repeat in one of two ways. Firstly the mosquito will be less likely to enter a house due to the excito-repellent effect of the insecticide on the nets, and secondly once it enters a house it will be repelled from a protected human due to the physical barrier of the net and the effects of the insecticide. Not all mosquitoes successfully feed upon entering a house even before the introduction of an intervention. Therefore the probability of repeating, feeding or dying needs to be relative to that seen in the absence of LLIN. The proportion repeating ($r_{N0}$), feeding successfully ($s_{N0}$) and dying ($d_{N0}$) in the presence of LLIN will therefore be,

$$\begin{matrix} r_{N0}=\left(1-\frac{k'_{1}}{k_{0}}\right)\left(\frac{j'_{1}}{j'_{1}+l'_{1}}\right) \\ s_{N0}=\frac{k'}{k_{0}} \\ d_{N0}=\left(1-\frac{k'_{1}}{k_{0}}\right)\left(\frac{l'_{1}}{j'_{1}+l'_{1}}\right) \end{matrix}$$

where $j'_{1}=\left(1-\frac{N_{1}}{N_{0}}\right)+\frac{N_{1}}{N_{0}}j_{1}$, $k'_{1}=\frac{N_{1}}{N_{0}}k_{1}$ and $l'_{1}=\frac{N_{1}}{N_{0}}l_{1}$.

## Mass Drug Administration (MDA) and Mass Screening and Treatment (MSAT)

Each round of mass drug administration is assumed to have an effectiveness that acts at the individual level. If the drug is effective then it is assumed to fully clear any existing infections and provide a period of prophylaxis. Let *d_P_* be the total duration of prophylaxis. If a person is infected then upon receiving an effective drug, they enter state T (see Protocol S1) of mean duration *d_T_,* and subsequently move to state P for mean duration *d_P_ –d_T_*. If they are in state S or P, then they enter state P for mean duration *d_P_*.

If they were in an infected state, the infectivity to mosquitoes while in state T varies depending on the infectivity of the state they were in at the time of treatment. The infectivity in T is modeled as being a constant multiple $\varsigma_{T}\in[0,1]$ of the infectivity of the previous state, where $\varsigma_{T}$ depends on the drug used.

Mass screening and treatment is modeled in the same way but in this case drugs are only given to those who have infection that is detectable via microscopy, that is, infection states *A* and *D*. The sensitivity of the diagnostic test is not explicitly incorporated: instead we assume that imperfect sensitivity is reflected by excluding infected individuals in state U from treatment.

## Pre-erythrocytic Vaccine

Pre-erthrocytic vaccines reduce the probability of liver-stage infection following sporozoite challenge. Phase II trial results from the RTS,S vaccine suggest a leaky vaccine which reduces the probability of infection rather than an all-or-nothing vaccine which would provide long-term protection for a subset of those vaccinated. Thus at time *t* after vaccination, at each infectious bite, the probability of infection is reduced to,

$b=\left(1-Ve^{-\gamma_{V}t}\right)b_{0}$,

where *V* is the vaccine efficacy, *γ_V_* is the efficacy decay rate and $b_{0}$ is the probability of infection given their natural anti-infection immunity.

It is not currently clear how, if at all, the RTS,S affects the natural development of immunity. Here we assume that natural anti-infection immunity increases with each infectious challenge just as it would in the absence of a vaccine. However, by reducing the probability of infection, our model assumes that the rate of acquisition of clinical immunity, which is assumed to be acquired only if blood-stage infection occurs, is also reduced.

## Correlations between Interventions

To explore the impact of non-random distribution of different interventions, we considered a range of correlations between who received a single intervention at each round and between different interventions at a single round.

Let the interventions under consideration be labelled by $j=1,...,n$. In the simulation model, each individual $i$has a vector $u_{i}$ of length $n$ which determines their propensity to take up each intervention. $u_{i}$ remains fixed over time, and is chosen from a multivariate normal distribution

$$u_{i}\sim MVN\left(u_{0},V\right)$$

When intervention $j$ is distributed at time $t$, choose a random variable for each individual $i$

$$z_{ijt}\sim N(u_{ij},1)$$

They then receive the intervention if and only if $z_{ijt}\leq0$.

The marginal standard deviations of the distribution of $u_{i}$ determine the extent to which for a single intervention, it is the same people who receive it at each round. The correlations between interventions are determined by the off-diagonal elements of $V$. Finally, the elements of $u_{0}$ determine the per-round coverage. These parameters are specified as follows.

First, for each intervention define the correlation $\rho_{j}$, $j=1,...,n$. $\rho_{j}=0$ means that a random choice of people is made at each round, whilst as $\rho_{j}$ approaches 1, the same people tend to receive intervention $j$ at every round. The marginal standard deviations of the distribution of $u_{i}$ are given by

$$\sigma_{j}=\sqrt{\frac{\rho_{j}}{1-\rho_{j}}}$$

Examples of how the probability of receiving a particular intervention varies beteen people are shown in Figure S2.3 for different values of $\rho_{j}$.

Second, for each $j,k=1,...,n, j\neq k$, specify the correlation between interventions, $-1\leq\rho_{jk}\leq1$, and put

$$\begin{matrix} V_{jj}=\sigma_{j}^{2} \\ V_{jk}=\sigma_{j}\sigma_{k}\rho_{jk}, j\neq k \end{matrix}$$

$\rho_{jk}=0$ means that receipt of different interventions are independent of each other, $\rho_{jk}=1$ means that the same people receive both, and $\rho_{jk}=-1$ means that people who receive one do not receive the other, to the extent that these are possible given the coverage of each intervention.

The marginal distribution of $z_{ijt}$, integrating over the values of $u_{ij}$, is

$$z_{ijt}\sim N(u_{0j},1+\sigma_{j}^{2})$$

and

$$P(z_{ijt}<0)=\Phi\left(\frac{-u_{0j}}{\sqrt{1+\sigma_{j}^{2}}}\right)$$

where $\Phi(.)$ is the standard normal cumulative distribution function. Hence in order to have a per-round coverage of $P_{j}$, we need to put

$$u_{0j}=-\Phi^{-1}(P_{j})\sqrt{1+\sigma_{j}^{2}}$$

where $\Phi^{-1}(.)$ denotes the inverse function of $\Phi(.)$.

For a pair of interventions with coverages of $P_{1}$ and $P_{2}$, the probability of receiving both in a single round of each is given by

$$P_{12}=\Phi_{2}\left(\Phi^{-1}(P_{1}), \Phi^{-1}(P_{2}), \rho_{12}\right)$$

where $\Phi_{2}\left(x, y, \rho_{12}\right)$ is the probability that $X\leq x$ and $Y\leq y$when $X$ and $Y$ have a bivariate normal distribution, with zero means, standard deviations of 1 and correlation $\rho_{12}$. This and the probability of receiving neither are plotted against $\rho_{12}$ for three pairs of $P_{1}$ and $P_{2}$ in Figure S2.4.

**Figure S2.3: Examples of the distribution between people of the probability of receiving a particular intervention for various correlations between distribution rounds. The overall per-round probability is 0.6 in each case.**

**Figure S2.4: Probability of receiving both or none of two interventions, plotted against the correlation between them for three pairs of values for the per-round coverage of each intervention, $P_{1}$ and** .

| 1. *Probability of receiving both* | 1. *Probability of receiving neither* |
| --- | --- |
|  | |

Except where specified, all the results assume no correlation between different interventions; whilst for each intervention it is assumed to be the same people receiving it at each round at which it is distributed, achieved by putting $\sigma_{j}=0.9999$.

## References

1. Le Menach A, Takala S, McKenzie FE, Perisse A, Harris A, et al. (2007) An elaborated feeding cycle model for reductions in vectorial capacity of night-biting mosquitoes by insecticide-treated nets. Malar J 6: 10.

2. Killeen GF, Ross A, Smith T (2006) Infectiousness of malaria-endemic human populations to vectors. Am J Trop Med Hyg 75: 38-45.

3. Killeen GF, Smith TA (2007) Exploring the contributions of bed nets, cattle, insecticides and excitorepellency to malaria control: a deterministic model of mosquito host-seeking behaviour and mortality. Trans R Soc Trop Med Hyg 101: 867-880.

4. Molineaux L, Shidrawi GR, Clarke JL, Boulzaguet JR, Ashkar TS (1979) Assessment of insecticidal impact on the malaria mosquito's vectorial capacity, from data on the man-biting rate and age-composition. Bull World Health Organ 57: 265-274.
