# Supplementary material for: Reducing Plasmodium falciparum Malaria Transmission in Africa: A Model-Based Evaluation of Intervention Strategies
Source: PLoS Med. 2010 Aug 10;7(8):e1000324. doi: 10.1371/journal.pmed.1000324 (PMC2919425; doi:10.1371/journal.pmed.1000324)
Supplement: Protocol S3 — Bayesian model fitting and parameter values. (0.92 MB DOC) [file pmed.1000324.s007.doc]

**Reducing *Plasmodium falciparum* malaria transmission in Africa: a model-based evaluation of intervention strategies**

Jamie T Griffin^1^, T. Deirdre Hollingsworth^1^, Lucy C Okell^1^, Thomas S Churcher^1^, Michael White^1^, Wes Hinsley^1^, Teun Bousema^2^, Chris J Drakeley^2^, Neil M Ferguson^1^, María-Gloria Basáñez^1^, Azra C Ghani^1^.

1. *MRC Centre for Outbreak Analysis & Modelling, Department of Infectious Disease Epidemiology, Imperial College London*
2. *Department of Infectious Diseases, London School of Hygiene & Tropical Medicine*

# PROTOCOL S3

**BAYESIAN MODEL FITTING & PARAMETER VALUES**

# Bayesian Model Fitting and Parameter Values

For model fitting we used the equilibrium solution to fit the compartmental model to the prevalence of parasitemia (by microscopy and/or PCR) and clinical disease incidence by age across transmission settings, assuming that these data can be approximated by a non-seasonal equilibrium. Model fitting was undertaken conditional on the EIR in the location and thus was not used to estimate vector parameters directly. EIR estimates from each location were used to construct informative priors for this parameter for each location. Bayesian Markov chain Monte Carlo (MCMC) methods were used for model fitting.

The four functional forms for infection-blocking immunity and clinical immunity, including the option of no immunity, (see Protocol S1) were considered in model fitting with the best model structure chosen as that with the largest posterior probability. Blood-stage anti-parasite immunity was assumed to be acquired only with age as it was not possible to distinguish exposure-driven parasite immunity from exposure-driven infection-blocking immunity from these data.

In order to reduce the number of parameters being fitted, the parameters which were reasonably precisely known from published sources were kept fixed. Rates of loss of immunity were also fixed, as there is no information about these in the data we have. As we are interested in this paper primarily in infection (parasitemia) and not clinical disease, immunity against infection has the greatest impact on our results. Hence in a sensitivity analysis we explored how results change with different values of the parameter *d*_B_ (see Protocol S5). We also fixed the parameters for the duration of patent infection with and without immunity during model fitting to overcome identifiability issues.

Table S3.1 lists the parameters considered in the fitting, their prior and fitted posterior distributions and associated source references.

**Table S3.1. Human Model Parameters and Prior Distributions**

| **Parameter Description** | **Symbol** | **Prior Distribution, with median and 95% interval if not fixed** | **Posterior Estimate and 95% Credible Interval, or fixed value** | **Units** | **References** |
| --- | --- | --- | --- | --- | --- |
| **Age and Heterogeneity** |  |  |  |  |  |
| Age-dependent biting parameter | $a_{0}$ | Fixed | 2920 | days | [1, 2] |
| Age-dependent biting parameter | $\rho$ | Fixed | 0.85 | - |
| Variance of log of heterogeneity in biting rates | $\sigma^{2}$ | Log-normal 1.62 (1.00, 2.61) | 1.27 (1.12, 1.44) | - | [3] |
| **Human Infectious Periods** |  |  |  |  |  |
| Latent period | *d_E_* | Fixed | 12 | days | [4] |
| Patent infection with no immunity (including disease) | *d_I_* | Fixed | 200 | days | [4, 5] |
| Sub-patent infection | *d_U_* | Weibull 67 (30, 100) | 168 (156, 181) | days | [5] |
| Prophylaxis with SP following treatment | *d_P_* | Fixed | 25 | days | [6] |
| Clinical disease with treatment^1^ | *d_T_* | Fixed | 5 | days | [7] |
| Clinical disease without treatment^2^ | *d_D_* | Fixed | 5 | days | [8] |
| **Infection Immunity** |  |  |  |  |  |
| Decay parameter | *d_B_* | Fixed | 10 | years | - |
| Scale parameter (see section 3.1.2) | *a_B_* | Gamma 8.39 (1.21, 27.9) | 11.24 (10.66, 11.84) | years | - |
| Shape parameter | *κ_B_* | Gamma 1.68 (0.24, 5.57) | 4.93 (4.36, 5.57) | - | - |
| Duration in state where immunity doesn’t increase following an initial exposure | $\gamma$ | 1/$\gamma$~Gamma  1685 (1.02, 1.7E16) | 69.5 (57.3, 84.3) | days | - |
| **Clinical Immunity** |  |  |  |  |  |
| Immunity level of newborn relative to mother | $P_{_{CM}}$ | Fixed | 0.5 | - | - |
| Decay parameter for maternal immunity | *d_M_* | Fixed | 255.5 | days | [9] |
| Decay parameter for acquired clinical immunity | *d_C_* | Fixed | 30 | years | [10] |
| Scale parameter (see section 3.1.2) | *a_C_* | Gamma 8.39 (1.21, 27.9) | 6.54 (6.28, 6.84) | years | - |
| Shape parameter | *κ_C_* | Gamma 1.68 (0.24, 5.57) | 4.13 (3.43, 4.93) | - | - |
| **Parasite clearance immunity** |  |  |  |  |  |
| Duration of patent infection with maximum immunity | *d_MIN_* | Fixed | 160 | days | Based on fitting to data from Garki project (see below) |
| Decay parameter | *d_A_* | Fixed | 10 | years | - |
| Scale parameter | *I_A_*_0_ | Fixed | 4732.5 | - | - |
| Shape parameter | *κ_A_* | Fixed | 5 | - | - |
| **Treatment** |  |  |  |  |  |
| Proportion treated | *f_T_* | Varied between transmission sites | Range 0.05 to 1 depending on transmission site | - | - |
| Infectivity | | | | | |
| Probability of human infection from an infectious bite with no immunity | *b_MAX_* | Beta 0.5 (0.09, 0.91) | 0.89 (0.78, 0.98) | - | [11-14] |
| Probability of human infection from an infectious bite with full immunity | *b_MIN_* | Beta 0.18 (0.03, 0.48) | 0.005 (0.002, 0.008) | - | - |
| Onward infectivity to mosquitoes (see section 3.1.3): |  |  |  |  |  |
| from treated state (with non-gametocytocidal drug) | *c_T_* | 0.10 (0.05, 0.17) | 0.12 | - | See below |
| from untreated disease | *c_D_* | 0.30 (0.16, 0.46) | 0.40 | - | See below |
| from patent infection | *c_A_* | 0.10 (0.05, 0.17) | 0.12 | - | See below |
| from sub-patent infection | *c_U_* | 0.005 (0.002, 0.009) | 0.02 | - | See below |
| Delay from emergence of blood-stage parasites to onward infectivity | *t_l_* | Fixed | 12.5 | days | [15] |

1. Most clinical trials show clearance of parasites by ~3 days. The value of 5 days was chosen to reflect imperfect adherence outside trial condition.
2. Note that this does not include the additional period of asymptomatic parasitaemia that follows an untreated infection.

## Notes on parameters

### Duration of stages of infection

Using the notation set out in Protocol S1, the rate of leaving the prophylactic state after treatment is given by $r_{P}=\frac{1}{d_{P}-d_{T}}$whilst the recovery rate from asymptomatic infection with no immunity is . Parameter vectors with $d_{P}\leq d_{T}$ or $d_{I}\leq d_{D}$were rejected in the MCMC sampling. The parameter *w_A_,* which determines how much $r_{A}$ increases with maximum anti-parasite immunity, is given by $w_{A}=\frac{1}{d_{MIN}r_{A0}}$.

### Rate of acquisition of Immunity

For model fitting, we parameterized the immunity functions in terms of *a_B_* and *a_C_* rather than *I_B_*_0_ and *I_C_*_0_ to reduce the dependency among the parameters. *I_B_*_0_ and *I_C_*_0_ are calculated as follows:

$$\begin{matrix} I_{B0}=\frac{\varepsilon_{0}d_{B}}{\varepsilon_{0}\gamma+1}\left(1+\rho/\left(d_{B}/a_{0}-1\right)\exp(-a_{B}/a_{0})-\left(1+\rho/\left(d_{B}/a_{0}-1\right)\right)\exp(-a_{B}/d_{B})\right) \\ I_{C0}=\Lambda_{0}d_{C}\left(1+\rho/\left(d_{C}/a_{0}-1\right)\exp(-a_{C}/a_{0})-\left(1+\rho/\left(d_{C}/a_{0}-1\right)\right)\exp(-a_{C}/d_{C})\right) \end{matrix}$$

where $\varepsilon_{0}=20/365$ and $\Lambda_{0}=\varepsilon_{0}b_{MAX}$, the daily EIR and force of infection with no immunity, if there is an annual EIR of 20 ibppy.

### Infectivity to mosquitoes

The parameters *c*_D_, *c*_T_, *c*_A_ and *c*_U_ were assigned the prior distributions given in Table S3.1, informed by the relationship between asexual parasite density and subsequent infectivity estimated in  [15]. The estimates were then updated by fitting the whole transmission cycle simultaneously to mosquito biting rate and human parasite prevalence data from the Garki project [16] and to data from four studies in which mosquitoes were fed on volunteers from malaria-endemic areas [17-20]. However, for the settings considered in this paper for which we had data on mosquito biting rates (those in Cameroon, Democratic Republic of Congo, Mozambique and Uganda), the EIRs implied by the model did not match the recorded values at those sites. So the four infectivity parameters were then rescaled, while keeping the relative infectivity of the different states constant, to fit the recorded EIRs. Further fitting of the whole transmission cycle in multiple settings with data on both mosquito biting rates and human parasite prevalence will be needed to properly validate the model.

## Summary of Parasite Prevalence and Clinical Incidence Data used for Model Fitting

### Parasite Prevalence Data

For model fitting we used age-stratified data on the prevalence of parasitemia as determined by microscopy and/or PCR. Using a previously published comprehensive literature review as a starting point [21, 22], we limited our fitting to data sources where:

1. The data were a random sample of individuals within the age-groups specified;
2. Sample sizes for prevalence estimates were reported;
3. Concurrent EIR estimates were available for use as prior distributions;

Table S3.2 summarises the data and sources used in model fitting.

**Table S3.2**. Summary of Parasite Prevalence Data used for Model Fitting

| **Country** | **Site** | **EIR** | **Age-standardized parasite prevalence in under 15s by microscopy** | **Age-stratification (groups)** | **Microscopy (M) / PCR (P)** | **Source References** |
| --- | --- | --- | --- | --- | --- | --- |
| Burkina Faso | Karangasso | 263 | 0.596 | 0-4, 5-9, 10-14 | M | [23] |
| Burkina Faso | Tago | 82 | 0.471 | 6 months – 15 | M | [24] |
| Cameroon | Ebolakounou | 17.7 | 0.615 | 0-5, 6-10,11-15, 16+ | M | [25] |
| Cameroon | Etoa | 511 | 0.555 | 0-6 months, 6 months-1, 1-5, 5-9, 10-15, 16+ | M | [26] |
| Cameroon | Koundou | 176 | 0.69 | 0-5, 6-10, 11-15,16+ | M | [25] |
| Cameroon | Mutengene, Molyko, Likok, Vasingi | 161 | Not full age range | 2-9 | M | [27] |
| Cameroon | Simbok | 566 | 0.605 | 0-6 months, 6 months-1, 1-5, 5-9, 10-15, 16+ | M | [26] |
| Gambia | Bwiam | 0.92 | Not full age range | 1-4 | M | [28] |
| Gambia | Dasilami a | 1.21 | Not full age range | 1-4 | M | [28] |
| Gambia | Jahally | 4.17 | Not full age range | 1-4 | M | [28] |
| Gambia | Kerewan | 0.44 | Not full age range | 1-4 | M | [28] |
| Gambia | Kulari | 7.75 | Not full age range | 1-4 | M | [28] |
| Gambia | Salikene | 1.94 | Not full age range | 1-4 | M | [28] |
| Gambia | SareAlpha | 11.15 | Not full age range | 1-4 | M | [28] |
| Gambia | Saruja | 5 | Not full age range | 1-4 | M | [28] |
| Gambia | Sibanor | 3.24 | Not full age range | 1-4 | M | [28] |
| Gambia | Sutukoba | 0.99 | Not full age range | 1-4 | M | [28] |
| Ghana | Kassena Nankana District | 418 | 0.583 | 0-6 months, 6 months-2, 2-3, 3-4, 4-5, 5-10, 10-15, 15-20, 20-30, 30-40, 40-50, 50-60, 60+ | M | [29, 30] |
| Ghana | Kassena Nankana District | 300 | 0.86 | 0-1, 1-3, 3-5, 1-10, 10-20, 20-40, 40-60, 60+ | M, P | [31] |
| Kenya | Kilifi Town | 1.5 | Not full age range | 1 to 4 | M | [32] |
| Kenya | Kisumu | 260 | 0.91 | 0-1, 1-6, 6-15, 15-40, 40+ | M, P | [33] |
| Kenya | Mumias | 46.7 | 0.513 | 0-1, 1-4, 5-9, 10-15 | M | [34] |
| Kenya | Saradidi | 237 | Not full age range | 6 months – 6 | M | [11] |
| Kenya | Sokoke | 8 | Not full age range | 1-4 | M | [32] |
| Mozambique | Manhica | 15 | 0.258 | 0-2, 2-4, 4-6, 6-8, 8-10, 10-20, 20-40, 40-60, 60+ | M, P | [35] |
| Mozambique | Matola | 53 | Wider age range | All | M | [36] |
| Senegal | Diohine / Kotiokh / Ngayokheme | 11.6 / 26.5 / 8 | Not full age range | 0-9 | M | [37, 38] |
| Tanzania | Kilimanjaro region (high altitude) | 0.142 | 0.030 | 1-45 (individual-level) | M | [40] |
| Tanzania | Kilimanjaro region (medium altitude) | 4.1 | 0.100 | 1-45 (individual-level) | M | [40] |
| Tanzania | Kilimanjaro region (low altitude) | 48 | 0.270 | 1-45 (individual-level) | M | [40] |
| Tanzania | Near Muheza | 380 | Not full age range | 1-6 | M | [41] |
| Tanzania | Tanga region (high altitude) | 0.178 | 0.169 | 1-45 (individual-level) | M | [40] |
| Tanzania | Tanga region (medium altitude) | 3.89 | 0.284 | 1-45 (individual-level) | M | [40] |
| Tanzania | Tanga region (low altitude) | 163 | 0.619 | 1-45 (individual-level) | M | [40] |

### Clinical Incidence Data

The model was simultaneously fitted to the clinical incidence data reported from longitudinal studies in Dielmo and Ndiop [42].

### Model Fits

The best fitting model was one in which infection-blocking immunity is acquired with exposure but limited to new infections (Model 3 in Protocol S1, Section 1.1.4) and in which clinical immunity is acquired with exposure. Parasite prevalence as measured using PCR, which we assume captures all of the sub-patent infections as well as patent infections and clinical disease, is substantially higher across all age-groups in the studies considered. Parasite prevalence as measured using PCR, which we assume captures all of the sub-patent infections as well as patent infections and clinical disease, is substantially higher across all age-groups in the studies considered. , Figure S3.2 and Figure S3.3 show the fits of the best fitting model structure to the parasite prevalence by age as measured using microscopy, PCR and clinical incidence data respectively. Note that the model is able to capture the age-peak-shift with decreasing transmission intensity and the decline in both clinical incidence and parasite rates at older age-groups. Parasite prevalence as measured using PCR, which we assume captures all of the sub-patent infections as well as patent infections and clinical disease, is substantially higher across all age-groups in the studies considered.

**Figure S3.1. Model Fits to Parasite Prevalence Data.** The fits are shown for the settings by decreasing reported EIR from highest in the top left to lowest in the bottom right, reading across rows. The x-axis is age (in years) and the y-axis is parasite prevalence (as proportion) measured by microscopy. Red lines show the model fit and the blue points and lines represent the measured parasite prevalence and associated 95% confidence intervals. The measured parasite prevalence is plotted at the mid-point of the age-group for which it was reported.

**Figure S3.2. Model Fits to Parasite Prevalence Data measured by PCR.** The fits are shown for the settings by decreasing reported EIR from highest on the left to lowest on the right, reading across rows. 95% confidence intervals are shown for reported parasite prevalence as measured by both microscopy and PCR. The measured parasite prevalence is plotted at the mid-point of the age-group for which it was reported.

**Figure S3.3. Model Fits to Clinical Incidence Data.** Left-hand graph: Dielmo, Senegal, EIR=200. Right-hand graph: Ndiop, Senegal, EIR=20. The x-axis is age (in years) and the y-axis is incidence of clinical malaria per year. The measured clinical incidence is plotted at the mid-point of the age-group for which it was reported.

###

### Fitted relationship between EIR and parasite prevalence in children

In determining the likely success of combined intervention programs in a given setting it is important to capture correctly the relationship between the force of infection, as determined by the EIR, and parasite prevalence across age groups, particularly if recommendations are made on the basis of parasite prevalence as an indicator of the intensity of transmission. Here we examine this relationship with reference to a previous study that characterized this using similar data. Our fitted model is shown in Figure 1B in the main text.

We used a subset of the data points used by Smith et al. (2005) [21] supplemented by a number of additional data points. Figure S3.4 shows that our subset of data captures the pattern and ranges originally reported in [21]. Thus the different modeled relationships do not reflect differences in data used for fitting.

**Figure S3.4. Data points used for fitting showing EIR against parasite prevalence in under 15 years.** Blue points are those used in our fitting and [21]; red are those used only in our fitting; and pink are those used only in [21].

However, a number of data points reported by [21] considered more narrow age-groups and as such were not representative of the 0-15 yr age-range. As a number of these were from younger age-groups, this inflates the prevalence at low EIRs. By correctly accounting for the different age groups in a fully age-structured model, we predict a different relationship compared to the original study in which parasite prevalence at low EIRs is substantially less.

**Figure S3.5. Relationship between EIR and parasite prevalence in under 15 year olds.** Solid blue line: fitted relationship as in Figure 1B of the main text; solid pink line: relationship fitted in [21]; circles: parasite prevalence estimates which are representative of the 0-15 year age range.

However, it should be noted that substantial measurement error in EIRs is likely at these levels. This is accounted for in our model fitting by using the measured EIR as a prior distribution. shows the relationship between the measured EIR and the posterior estimate of the EIR across the settings. There is a close correlation between the estimates.

**Figure S3.6. Posterior estimate of the EIR plotted against the measured EIR in the 34 settings.**

Finally, Figure S3.7 shows the fitted relationship between EIR and parasite prevalence in children under 15 years where the sample is representative of this age-group. Here we plot the posterior EIR estimates, which are our revised estimate of the true EIR in these settings.

**Figure S3.7. Relationship between posterior estimate of the EIR and parasite prevalence.** Solid line: fitted model; Circles: data points using posterior estimate of the EIR rather than measured EIR.

## Vector Parameters

Parameters for *Anopheles* vectors were not fitted apart from the emergence rate over calendar time for the six settings as detailed in Protocol S4. A detailed literature review was undertaken to specify biologically realistic parameters for each of the three vector species considered. This is summarized in Table S3.3. It is assumed that the entomological parameters are the same for each species across different geographical locations. However, care should be taken when comparing the parameter estimates as they may vary between different vector populations, for example between the different forms of *An. gambiae s.s.* [43] (though see [44]) or depending on the number of alternative blood hosts [45]. Parameters such as the EIP within the mosquito are known to vary with climate [46], but for simplicity values are kept constant between locations and over time. Model predictions assume that none of the vector parameters change over the duration of the control intervention, other than those such as the death rate which are affected by anti-vector interventions.

**Table S3.3. Summary of Parameters for the Vector Component of the Transmission Model.** Superscript $v$is used to differentiate between vector species. Where possible, point estimates were taken from studies which investigated all three vector species to reduce study variability.

| **Definition** | **Model**  **parameter** | **Best estimate**  **with observed range / alternative values where available** | | | **References** |
| --- | --- | --- | --- | --- | --- |
|  |  | ***An. funestus*** | ***An. arabiensis*** | ***An. gambiae s.s.*** |  |
| Mean life expectancy (days) | $1/\mu_{0}$ | 8.9  (5.6-10.2) | 7.6^i^  (4.1-16.1) | 7.6  (4.5-16.1) | [47-53] |
| Mean duration between two consecutive blood meals (days)^ii^ | $\delta_{1}+\delta_{2}$ | 3 | 3 | 3 | [36, 54]^ii^ |
| Mean time spent foraging for a blood meal (days) | $\delta_{1}$ | 0.68 | 0.68 | 0.68 | [55] |
| Proportion of blood meals taken on humans prior to intervention (Human Blood Index) | $Q_{0}$ | 0.94  (0.73-0.96) | 0.71  (0.66-0.83) | 0.92  (0.86-1.0) | [56-60] |
| Endophily (proportion of mosquitoes resting indoors after feeding with no intervention) | $\chi$ | 0.86  (0.86,1.0) | 0.16  (0.16,0.49) | 0.86  (0.49,0.86) | [60-62] |
| Proportion of bites taken on humans whilst they are indoors ^iii^ | $\Phi_{I}$ | 0.98 | 0.96 | 0.97 | [63, 64] ^iii^ |
| Proportion of bites taken on humans whilst they are in bed ^iii^ | $\Phi_{B}$ | 0.90 | 0.90 | 0.89 | [63, 64] ^iii^ |
| Extrinsic incubation period (days) | $\tau_{M}$ | 10 | 10 | 10 | [65] |

Best estimates all taken from Tanzania for comparability (averaged over different seasons). Assumed to be constant for between *An. gambiae s.s.* and *An. arabiensis.*

Values ranging from 2 to 3 were given by [3] but assumed by original authors.

Were estimated using human behavior from Killeen et al. (2006) [64] and the vector biting patterns from Githeko et al. (1996) [63]. Though these studies were undertaken in different geographical locations, Githeko et al. (1996) [63] was the only study to have biting information for each of the three vector species under investigation. Biting rates and proportion of humans in bed were measured per hour. It is assumed all human hosts are indoors 1 hour before getting into bed and ½ hour after getting out of bed.

## Intervention Parameters

### LLINs and IRS

The assumed parameters for the LLIN/IRS interventions are summarized in Table S3.4 below with alternative estimates from the literature given in brackets. It is assumed that the parameters remain constant for the duration of the control program.

### **Table S3.4. Parameters for the effectiveness of LLINs and IRS**

| **Definition** | **Model**  **parameter** | **Best estimate with alternative estimates where available in brackets** | | | **References** |
| --- | --- | --- | --- | --- | --- |
|  |  | ***An. funestus*** | ***An. arabiensis*** | ***An. gambiae s.s.*** |  |
| Cycle repeating probability for LLINs | $r_{N0}$ | 0.56 (0.69) | 0.48 (0.23) | 0.56 (0.52) | [66-68] |
| Successful feeding with LLINs | $s_{N0}$ | 0.03 (0.14) | 0.39 (0.33) | 0.03 (0.16) | [66-68] |
| Insecticide mortality probability for LLINs | $d_{N0}$ | 0.41 (0.17) | 0.13 (0.44) | 0.41 (0.32) | [66-68] |
| Baseline repeating action for LLINs ^iv^ | $r_{NM}$ | 0.24 | 0.10 | 0.24 | [66, 67] |
| Cycle repeating probability for IRS with DDT | $r_{S0}$ | 0.63 | 0.60 | 0.60 | [69] |
| Cycle repeating probability for lambdacyhalothrin ^v^ | $r_{S0}$ | 0.207 | | | [70] |
| Half-life of LLIN efficacy | Log(2)/$\gamma_{N}$ | 2.64 years | | | [71] |
| Half-life of IRS DDT efficacy | Log(2)/$\gamma_{S}$ | 0.5 years | | | [72] |
| Half-life of IRS lambdacyhalothrin efficacy | Log(2)/$\gamma_{S}$ | 0.13 years | | | [70] |

Based on untreated net with 6 holes.

Assumed same value for all mosquito species.

### ACT for MDA/MSAT

ACT used in MDA or MSAT is assumed to fully cure existing infections (i.e. we assume no resistance). We assume that the relative infectiousness of an individual after receiving ACT is reduced by a factor $\varsigma_{T}$ compared to their pre-treatment infectious state. This value was assumed to be 0.154 based on previous modeling work informed by ACT trial data [73, 74]. The period of prophylaxis is assumed to be 25 days, based on the assumption that an artemisinin would be combined with a partner drug with a longer prophylactic time, such as SP.

## References

1. Carnevale, P., J.L. Frezil, M.F. Bosseno, F. Le Pont, and J. Lancien, *[The aggressiveness of Anopheles gambiae A in relation to the age and sex of the human subjects].* Bull World Health Organ, 1978. **56**(1): p. 147-54.

2. Port, G.R., P.F.L. Boreham, and J.H. Bryan, *The Relationship of Host Size to Feeding by Mosquitos of the Anopheles-Gambiae Giles Complex (Diptera, Culicidae).* Bulletin of Entomological Research, 1980. **70**(1): p. 133-144.

3. Smith, T., J.D. Charlwood, W. Takken, M. Tanner, and D.J. Spiegelhalter, *Mapping the densities of malaria vectors within a single village.* Acta Trop, 1995. **59**(1): p. 1-18.

4. Eyles, D.E. and M.D. Young, *The duration of untreated or inadequately treated Plasmodium falciparum infections in the human host.* J Natl Malar Soc, 1951. **10**(4): p. 327-336.

5. Falk, N., N. Maire, W. Sama, S. Owusu-Agyei, T. Smith, H.P. Beck, et al., *Comparison of PCR-RFLP and Genescan-based genotyping for analyzing infection dynamics of Plasmodium falciparum.* Am J Trop Med Hyg, 2006. **74**(6): p. 944-50.

6. Watkins, W.M., E.K. Mberu, P.A. Winstanley, and C.V. Plowe, *The efficacy of antifolate antimalarial combinations in Africa: a predictive model based on pharmacodynamic and pharmacokinetic analyses.* Parasitology Today, 1997. **13**(12): p. 459-464.

7. Zwang, J., E.A. Ashley, C. Karema, U. D'Alessandro, F. Smithuis, G. Dorsey, et al., *Safety and efficacy of dihydroartemisinin-piperaquine in falciparum malaria: a prospective multi-centre individual patient data analysis.* PLoS One, 2009. **4**(7): p. e6358.

8. Miller, M.J., *Observations on the natural history of malaria in the semi-resistant West African.* Trans R Soc Trop Med Hyg, 1958. **52**(2): p. 152-68.

9. Sehgal, V.M., W.A. Siddjiqui, and M.P. Alpers, *A seroepidemiological study to evaluate the role of passive maternal immunity to malaria in infants.* Trans R Soc Trop Med Hyg, 1989. **83 Suppl**: p. 105-6.

10. Deloron, P. and C. Chougnet, *Is immunity to malaria really short-lived?* Parasitol Today, 1992. **8**(11): p. 375-8.

11. Beier, J.C., C.N. Oster, F.K. Onyango, J.D. Bales, J.A. Sherwood, P.V. Perkins, et al., *Plasmodium falciparum incidence relative to entomologic inoculation rates at a site proposed for testing malaria vaccines in western Kenya.* Am J Trop Med Hyg, 1994. **50**(5): p. 529-36.

12. Dietz, K., L. Molineaux, and A. Thomas, *A malaria model tested in the African savannah.* Bull World Health Organ, 1974. **50**(3-4): p. 347-57.

13. Pull, J.H. and B. Grab, *A simple epidemiological model for evaluating the malaria inoculation rate and the risk of infection in infants.* Bull World Health Organ, 1974. **51**(5): p. 507-16.

14. Rickman, L.S., T.R. Jones, G.W. Long, S. Paparello, I. Schneider, C.F. Paul, et al., *Plasmodium falciparum-infected Anopheles stephensi inconsistently transmit malaria to humans.* Am J Trop Med Hyg, 1990. **43**(5): p. 441-5.

15. Ross, A., G. Killeen, and T. Smith, *Relationships between host infectivity to mosquitoes and asexual parasite density in Plasmodium falciparum.* Am J Trop Med Hyg, 2006. **75**(2 Suppl): p. 32-7.

16. Molineaux, L. and G. Gramiccia, *The Garki Project*. 1980, Geneva: World Health Organisation.

17. Bonnet, S., L.C. Gouagna, R.E. Paul, I. Safeukui, J.Y. Meunier, and C. Boudin, *Estimation of malaria transmission from humans to mosquitoes in two neighbouring villages in south Cameroon: evaluation and comparison of several indices.* Transactions of the Royal Society of Tropical Medicine and Hygiene, 2003. **97**(1): p. 53-59.

18. Boudin, C., M. Olivier, J.-F. Molez, J.-P. Chiron, and P. Ambroise-Thomas, *High Human Malarial Infectivity to Laboratory-Bred Anopheles gambiae in a Village in Burkina Faso.* Am J Trop Med Hyg, 1993. **48**(5): p. 700-706.

19. Githeko, A.K., A.D. Brandling-Bennett, M. Beier, F. Atieli, M. Owaga, and F.H. Collins, *The reservoir of Plasmodium falciparum malaria in a holoendemic area of western Kenya.* Transactions of the Royal Society of Tropical Medicine and Hygiene, 1992. **86**(4): p. 355-358.

20. Muirhead-Thomson, R.C., *The Malarial Infectivity of an African Village Population to Mosquitoes (Anopheles Gambiae): A Random Xenodiagnostic Survey.* Am J Trop Med Hyg, 1957. **6**(6): p. 971-979.

21. Smith, D.L., J. Dushoff, R.W. Snow, and S.I. Hay, *The entomological inoculation rate and Plasmodium falciparum infection in African children.* Nature, 2005. **438**(7067): p. 492-5.

22. Hay, S.I., C.A. Guerra, A.J. Tatem, P.M. Atkinson, and R.W. Snow, *Urbanization, malaria transmission and disease burden in Africa.* Nat Rev Microbiol, 2005. **3**(1): p. 81-90.

23. Boudin, C., V. Robert, J.P. Verhave, P. Carnevale, and P. Ambroise-Thomas, *Plasmodium falciparum and P. malariae epidemiology in a West African village.* Bull World Health Organ, 1991. **69**(2): p. 199-205.

24. Gazin, P., V. Robert, M. Cot, and P. Carnevale, *Plasmodium falciparum incidence and patency in a high seasonal transmission area of Burkina Faso.* Trans R Soc Trop Med Hyg, 1988. **82**(1): p. 50-5.

25. Bonnet, S., R.E. Paul, C. Gouagna, I. Safeukui, J.Y. Meunier, R. Gounoue, et al., *Level and dynamics of malaria transmission and morbidity in an equatorial area of South Cameroon.* Trop Med Int Health, 2002. **7**(3): p. 249-56.

26. Quakyi, I.A., R.G. Leke, R. Befidi-Mengue, M. Tsafack, D. Bomba-Nkolo, L. Manga, et al., *The epidemiology of Plasmodium falciparum malaria in two Cameroonian villages: Simbok and Etoa.* Am J Trop Med Hyg, 2000. **63**(5-6): p. 222-30.

27. Wanji, S., T. Tanke, S.N. Atanga, C. Ajonina, T. Nicholas, and D. Fontenille, *Anopheles species of the mount Cameroon region: biting habits, feeding behaviour and entomological inoculation rates.* Trop Med Int Health, 2003. **8**(7): p. 643-9.

28. Thomson, M.C., U. D'Alessandro, S. Bennett, S.J. Connor, P. Langerock, M. Jawara, et al., *Malaria prevalence is inversely related to vector density in The Gambia, West Africa.* Trans R Soc Trop Med Hyg, 1994. **88**(6): p. 638-43.

29. Koram, K.A., S. Owusu-Agyei, D.J. Fryauff, F. Anto, F. Atuguba, A. Hodgson, et al., *Seasonal profiles of malaria infection, anaemia, and bednet use among age groups and communities in northern Ghana.* Trop Med Int Health, 2003. **8**(9): p. 793-802.

30. Appawu, M., S. Owusu-Agyei, S. Dadzie, V. Asoala, F. Anto, K. Koram, et al., *Malaria transmission dynamics at a site in northern Ghana proposed for testing malaria vaccines.* Trop Med Int Health, 2004. **9**(1): p. 164-70.

31. Owusu-Agyei, S., K.P. Asante, M. Adjuik, G. Adjei, E. Awini, M. Adams, et al., *Epidemiology of malaria in the forest-savanna transitional zone of Ghana.* Malar J, 2009. **8**: p. 220.

32. Mbogo, C.N., R.W. Snow, E.W. Kabiru, J.H. Ouma, J.I. Githure, K. Marsh, et al., *Low-level Plasmodium falciparum transmission and the incidence of severe malaria infections on the Kenyan coast.* Am J Trop Med Hyg, 1993. **49**(2): p. 245-53.

33. Ofulla, A.V., A.M. Moormann, P.E. Embury, J.W. Kazura, P.O. Sumba, and C.C. John, *Age-related differences in the detection of Plasmodium falciparum infection by PCR and microscopy, in an area of Kenya with holo-endemic malaria.* Ann Trop Med Parasitol, 2005. **99**(4): p. 431-5.

34. Shililu, J.I., W.A. Maier, H.M. Seitz, and A.S. Orago, *Seasonal density, sporozoite rates and entomological inoculation rates of Anopheles gambiae and Anopheles funestus in a high-altitude sugarcane growing zone in Western Kenya.* Trop Med Int Health, 1998. **3**(9): p. 706-10.

35. Mayor, A., J.J. Aponte, C. Fogg, F. Saute, B. Greenwood, M. Dgedge, et al., *The epidemiology of malaria in adults in a rural area of southern Mozambique.* Malar J, 2007. **6**: p. 3.

36. Mendis, C., J.L. Jacobsen, A. Gamage-Mendis, E. Bule, M. Dgedge, R. Thompson, et al., *Anopheles arabiensis and An. funestus are equally important vectors of malaria in Matola coastal suburb of Maputo, southern Mozambique.* Med Vet Entomol, 2000. **14**(2): p. 171-80.

37. Robert, V., H. Dieng, L. Lochouran, S.F. Traore, J.F. Trape, F. Simondon, et al., *[Malaria transmission in the rural zone of Niakhar, Senegal].* Trop Med Int Health, 1998. **3**(8): p. 667-77.

38. Sokhna, C.S., F.B.K. Faye, A. Spiegel, H. Dieng, and J.F. Trape, *Rapid reappearance of Plasmodium falciparum after drug treatment among Senegalese adults exposed to moderate seasonal transmission.* Am J Trop Med Hyg, 2001. **65**(3): p. 167-70.

39. Charlwood, J.D., T. Smith, E. Lyimo, A.Y. Kitua, H. Masanja, M. Booth, et al., *Incidence of Plasmodium falciparum infection in infants in relation to exposure to sporozoite-infected anophelines.* Am J Trop Med Hyg, 1998. **59**(2): p. 243-51.

40. Drakeley, C.J., I. Carneiro, H. Reyburn, R. Malima, J.P. Lusingu, J. Cox, et al., *Altitude-dependent and -independent variations in Plasmodium falciparum prevalence in northeastern Tanzania.* J Infect Dis, 2005. **191**(10): p. 1589-98.

41. Curtis, C.F., C.A. Maxwell, R.J. Finch, and K.J. Njunwa, *A comparison of use of a pyrethroid either for house spraying or for bednet treatment against malaria vectors.* Trop Med Int Health, 1998. **3**(8): p. 619-31.

42. Trape, J.F. and C. Rogier, *Combating malaria morbidity and mortality by reducing transmission.* Parasitology Today, 1996. **12**(6): p. 236-240.

43. Lehmann, T. and A. Diabate, *The molecular forms of Anopheles gambiae: a phenotypic perspective.* Infect Genet Evol, 2008. **8**(5): p. 737-46.

44. Braimah, N., C. Drakeley, E. Kweka, F. Mosha, M. Helinski, H. Pates, et al., *Tests of bednet traps (Mbita traps) for monitoring mosquito populations and time of biting in Tanzania and possible impact of prolonged ITN use.* Int J Trop Insect Sci, 2005. **25**(3): p. 208-213.

45. Killeen, G.F., F.E. McKenzie, B.D. Foy, C. Bogh, and J.C. Beier, *The availability of potential hosts as a determinant of feeding behaviours and malaria transmission by African mosquito populations.* Trans R Soc Trop Med Hyg, 2001. **95**(5): p. 469-76.

46. Paaijmans, K.P., A.F. Read, and M.B. Thomas, *Understanding the link between malaria risk and climate.* Proc Natl Acad Sci U S A, 2009. **106**(33): p. 13844-9.

47. Garrett-Jones, C. and G.R. Shidrawi, *Malaria vectorial capacity of a population of Anopheles gambiae: an exercise in epidemiological entomology.* Bull World Health Organ, 1969. **40**(4): p. 531-45.

48. Costantini, C., S.G. Li, A. Della Torre, N. Sagnon, M. Coluzzi, and C.E. Taylor, *Density, survival and dispersal of Anopheles gambiae complex mosquitoes in a west African Sudan savanna village.* Med Vet Entomol, 1996. **10**(3): p. 203-19.

49. Garrett-Jones, C. and B. Grab, *The Assessment of Insecticidal Impact on the Malaria Mosquito's Vectorial Capacity, from Data on the Proportion of Parous Females.* Bull World Health Organ, 1964. **31**: p. 71-86.

50. Gillies, M.T., *Observations on Nulliparous and Parous Rates in Some Common East African Mosquitoes.* Ann Trop Med Parasitol, 1963. **57**: p. 435-42.

51. Gillies, M.T. and T.J. Wilkes, *A study of the age-composition of populations of Anopheles gambiae Giles and A. funestus Giles in North-Eastern Tanzania.* Bull Entomol Res, 1965. **56**(2): p. 237-62.

52. Krafsur, E.S. and C. Garrett-Jones, *The survival in nature of Wuchereia-infected Anopheles funestus Giles in North-eastern Tanzania.* Trans R Soc Trop Med Hyg, 1977. **71**(2): p. 155-60.

53. Appawu, M.A., *Genetic diversity of Anopheles vectors and transmission of malaria in the Kassena-Nankana District, Ghana*, in *Noguchi Memorial Institute for Medical Research*

University of Ghana.

54. Killeen, G.F., F.E. McKenzie, B.D. Foy, C. Schieffelin, P.F. Billingsley, and J.C. Beier, *A simplified model for predicting malaria entomologic inoculation rates based on entomologic and parasitologic parameters relevant to control.* Am J Trop Med Hyg, 2000. **62**(5): p. 535-44.

55. Killeen, G.F. and T.A. Smith, *Exploring the contributions of bed nets, cattle, insecticides and excitorepellency to malaria control: a deterministic model of mosquito host-seeking behaviour and mortality.* Trans R Soc Trop Med Hyg, 2007. **101**(9): p. 867-80.

56. Dabire, K.R., A. Diabate, L. Pare-Toe, J. Rouamba, A. Ouari, D. Fontenille, et al., *Year to year and seasonal variations in vector bionomics and malaria transmission in a humid savannah village in west Burkina Faso.* J Vector Ecol, 2008. **33**(1): p. 70-5.

57. Dia, I., T. Diop, I. Rakotoarivony, P. Kengne, and D. Fontenille, *Bionomics of Anopheles gambiae Giles, An. arabiensis Patton, An. funestus Giles and An. nili (Theobald) (Diptera: Culicidae) and transmission of Plasmodium falciparum in a Sudano-Guinean zone (Ngari, Senegal).* J Med Entomol, 2003. **40**(3): p. 279-83.

58. Ndiath, M.O., C. Brengues, L. Konate, C. Sokhna, C. Boudin, J.F. Trape, et al., *Dynamics of transmission of Plasmodium falciparum by Anopheles arabiensis and the molecular forms M and S of Anopheles gambiae in Dielmo, Senegal.* Malar J, 2008. **7**: p. 136.

59. Marrama, L., R. Jambou, I. Rakotoarivony, J.M. Leong Pock Tsi, J.B. Duchemin, S. Laventure, et al., *Malaria transmission in Southern Madagascar: influence of the environment and hydro-agricultural works in sub-arid and humid regions. Part 1. Entomological investigations.* Acta Trop, 2004. **89**(2): p. 193-203.

60. Tirados, I., C. Costantini, G. Gibson, and S.J. Torr, *Blood-feeding behaviour of the malarial mosquito Anopheles arabiensis: implications for vector control.* Med Vet Entomol, 2006. **20**(4): p. 425-37.

61. Molineaux, L., G.R. Shidrawi, J.L. Clarke, J.R. Boulzaguet, and T.S. Ashkar, *Assessment of insecticidal impact on the malaria mosquito's vectorial capacity, from data on the man-biting rate and age-composition.* Bull World Health Organ, 1979. **57**(2): p. 265-74.

62. Oyewole, I.O., T.S. Awolola, C.A. Ibidapo, A.O. Oduola, O.O. Okwa, and J.A. Obansa, *Behaviour and population dynamics of the major anopheline vectors in a malaria endemic area in southern Nigeria.* J Vector Borne Dis, 2007. **44**(1): p. 56-64.

63. Githeko, A.K., M.W. Service, C.M. Mbogo, and F.K. Atieli, *Resting behaviour, ecology and genetics of malaria vectors in large scale agricultural areas of Western Kenya.* Parassitologia, 1996. **38**(3): p. 481-9.

64. Killeen, G.F., J. Kihonda, E. Lyimo, F.R. Oketch, M.E. Kotas, E. Mathenge, et al., *Quantifying behavioural interactions between humans and mosquitoes: evaluating the protective efficacy of insecticidal nets against malaria transmission in rural Tanzania.* BMC Infect Dis, 2006. **6**: p. 161.

65. Gu, W., C.M. Mbogo, J.I. Githure, J.L. Regens, G.F. Killeen, C.M. Swalm, et al., *Low recovery rates stabilize malaria endemicity in areas of low transmission in coastal Kenya.* Acta Trop, 2003. **86**(1): p. 71-81.

66. Curtis, C.F., J. Myamba, and T.J. Wilkes, *Comparison of different insecticides and fabrics for anti-mosquito bednets and curtains.* Med Vet Entomol, 1996. **10**(1): p. 1-11.

67. Lines, J.D., J. Myamba, and C.F. Curtis, *Experimental hut trials of permethrin-impregnated mosquito nets and eave curtains against malaria vectors in Tanzania.* Med Vet Entomol, 1987. **1**(1): p. 37-51.

68. Mathenge, E.M., J.E. Gimnig, M. Kolczak, M. Ombok, L.W. Irungu, and W.A. Hawley, *Effect of permethrin-impregnated nets on exiting behavior, blood feeding success, and time of feeding of malaria mosquitoes (Diptera: Culicidae) in western Kenya.* J Med Entomol, 2001. **38**(4): p. 531-6.

69. Smith, A. and D.J. Webley, *A verandah-trap hut for studying the house-frequenting habits of mosquitoes and for assessing insecticides. 3. The effect of DDT on behavior and mortality.* Bull Entomol Res, 1969. **59**(1): p. 33-46.

70. N'Guessan, R., V. Corbel, M. Akogbeto, and M. Rowland, *Reduced efficacy of insecticide-treated nets and indoor residual spraying for malaria control in pyrethroid resistance area, Benin.* Emerg Infect Dis, 2007. **13**(2): p. 199-206.

71. Mahama, T., E.J. Desiree, C. Pierre, and C. Fabrice, *Effectiveness of permanet in Cote d'Ivoire rural areas and residual activity on a knockdown-resistant strain of Anopheles gambiae.* J Med Entomol, 2007. **44**(3): p. 498-502.

72. World Health Organisation, *Pesticides and their application*. 2006, World Health Organisation, : Geneva.

73. Okell, L.C., C.J. Drakeley, T. Bousema, C.J. Whitty, and A.C. Ghani, *Modelling the impact of artemisinin combination therapy and long-acting treatments on malaria transmission intensity.* PLoS Med, 2008. **5**(11): p. e226; discussion e226.

74. Okell, L.C., C.J. Drakeley, A.C. Ghani, T. Bousema, and C.J. Sutherland, *Reduction of transmission from malaria patients by artemisinin combination therapies: a pooled analysis of six randomized trials.* Malar J, 2008. **7**: p. 125.
