# Supplementary material for: Reducing Plasmodium falciparum Malaria Transmission in Africa: A Model-Based Evaluation of Intervention Strategies
Source: PLoS Med. 2010 Aug 10;7(8):e1000324. doi: 10.1371/journal.pmed.1000324 (PMC2919425; doi:10.1371/journal.pmed.1000324)
Supplement: Protocol S4 — Seasonal patterns and transmission settings. (0.49 MB DOC) [file pmed.1000324.s008.doc]

**Reducing *Plasmodium falciparum* malaria transmission in Africa: a model-based evaluation of intervention strategies**

Jamie T Griffin^1^, T. Deirdre Hollingsworth^1^, Lucy C Okell^1^, Thomas S Churcher^1^, Michael White^1^, Wes Hinsley^1^, Teun Bousema^2^, Chris J Drakeley^2^, Neil M Ferguson^1^, María-Gloria Basáñez^1^, Azra C Ghani^1^.

1. *MRC Centre for Outbreak Analysis & Modelling, Department of Infectious Disease Epidemiology, Imperial College London*
2. *Department of Infectious Diseases, London School of Hygiene & Tropical Medicine*

# PROTOCOL S4

**SEASONAL PATTERNS & TRANSMISSION SETTINGS**

# Seasonal patterns

Seasonality of climate results in different mosquito and malarial dynamics across Africa. To investigate the effect of this seasonality on the effectiveness of interventions, we chose to study six example settings. In each setting, a seasonal, vector-specific birth rate was fitted to one or two year’s field data on biting rates or EIR by species.

## Seasonally varying mosquito birth rate

For each species in each site, the seasonal curve for the birth rate took the following parametric form:

$$\beta(t)=m\mu g\left(\theta(t)\right)$$

Here *m* is the mean density of mosquitoes per person over the year, *μ*  is the death rate and $g(\theta(t))$ is the normalized birth rate:

$$g(\theta)=\frac{2\pi f(\theta)}{\int_{0}^{2\pi} f(\theta) d\theta}$$

The non-normalized birth rate is given by the curve

$$f(\theta)=c+(1-c)\left(\frac{1+\cos(\theta)}{2}\right)^{\kappa}$$

where *t* is the date in days from the start of the year, and $\theta=2\pi(t/365-u)$ . Thus $u\in[0,1]$ positions the peak of the seasonal curve and $c\in[0,1]$, $\kappa>0$ give the height and shape of the seasonal curve respectively.

The parameters determining the birth rate, $c$, $\theta$ and $\kappa$ were estimated by fitting the transmission model with this birth rate formulation to monthly reported EIRs or biting rate measurements using least squares on the square root-transformed data. For the fitting the other model parameters were fixed at the values estimated previously.

## Transmission Settings

### Kassena-Nankana District, Ghana

Kassena-Nankana District is a rural area in Northern Ghana which experiences intense but highly seasonal malaria transmission. High levels of infant and child mortality are associated with *P. falciparum* malaria in this area, with an estimated 23% of child deaths in 1991-92 attributed to malaria infection [1].

The area experiences a single short wet season between May and October and a long dry season from November to April with an average annual rainfall of 850 mm [2,3]. Clinical disease and malaria-associated mortality patterns closely follow the seasonal transmission patterns, with peaks observed in incidence between June and October [1,4,5]. Prevalence of parasitemia peaks in infants and children with levels of 50-80% [4]. Prevalence of parasitemia also appears to vary seasonally, with significantly higher prevalence during the transmission season (61% in the general population) than during the dry season (22% in the general population) [4].

Two main vector species – *An. gambiae s.s*. and *An. funestus* - account for the majority of transmission with *An. gambiae s.s*. dominating during the peak rainy season and lower levels of transmission via *An. funestus* occurring throughout the year [6].

There are three main ecosystems in this region [6]. Here we study the irrigated area where malaria transmission is highest. The annual EIR is estimated to be 630 ibppy [6].

**Figure S4.1. Fit of seasonality curve to EIR data from KND, Ghana. Markers: adapted from data presented by Appawu et al. [6] for irrigated area in Navrongo. Solid lines are fitted daily EIR.**

### Nkoteng, Cameroon

The village of Nkoteng is located in the central forest-savannah of Cameroon. In this equatorial region there are two rainy seasons – March to June and September to November. There are few malaria studies in this region, but transmission rates are high, with malaria prevalence estimated to be greater than 50% [7]. Annual rainfall in the study period of 1999-2000 was 1466mm, and the average temperature was in the range 23-27°C [8]. The majority species of anopheles is *An. funestus* accounting for 91% of mosquito catches. The remaining mosquitoes were almost exclusively *An. gambiae s.l.*, of which the vast majority of those tested (97%) were *An. gambiae s.s.* [8]. Both species were present throughout the year, but with some variation through the year. The overall indoor human biting rate, averaged over the period of study, was 3.4 bites per person per night for *An. funestus* and 0.4 for *An. gambiae s.l.* The indoor biting rate for *An.* *funestus* varied slightly through the year, being lowest at the end of the dry season, in February.

**Figure S4.2 – Fit of seasonality curve to biting data from Nkoteng, Cameroon. Dots: data adapted from Cohuet et al. (2004) [8] showing bites per person per night measured over a 2-year period. Lines show the fitted curves.**

Note that there were substantial differences between the two years in the measured bites per night by *An.funestus* and hence the fitted curve is an average for these years.

### Kinkolé, Democratic Republic of the Congo

The village of Kinkolé, on the Bateké plateau 60 km east of Kinshasa, had approximately 2,900 inhabitants in 1991, when a bednet trial was conducted in this and two neighbouring villages [9]. We used baseline data, prior to the distribution of bednets. The climate is characterized by a long wet season from October to May, with a short dry season in February and March and a longer dry season from June to September. Temperatures ranged from 25 to 27.5°C. *Plasmodium falciparum* prevalence was 63.6% at the start of the study period. *An. gambiae s.l.* formed 97% of the mosquitoes captured in Kinkolé. Since PCR was not performed on these mosquitoes, we used previous studies to motivate an assumption that *An. arabiensis* was not present [10] and that all the mosquitoes had the characteristics of *An. gambiae s.s.*

**Figure S4.3 – Fit of seasonality curve to biting data from Kinkole, DRC. Dots: data adapted from Karch et al. (1993)** **[9] showing bites per person per night. Lines show the fitted curves.**

### Matola, Maputo, Mozambique

The climate of Maputo, Mozambique alternates between a hot rainy season from November to April and a cool dry season for the remainder of the year [11]. Mean annual rainfall is 650-850mm, and temperatures range from 14-30°C. Matola is a suburb of Maputo which was partially populated by displaced people during the study period of November 1994 to April 1996. Malaria prevalence ranged from 33-63%, dominated by *P. falciparum.* Mosquitoes breed in saltmarsh and freshwater pools in this coastal suburb. *An. funestus* was present throughout the year (46% of mosquitoes caught), whereas *An. arabiensis (*the second most prevalent species) was concentrated in the rainy season.

**Figure S4.4 – Fit of seasonality curve to biting data from Maputo, Mozambique. Dots: data adapted from Mendis et al. (2000)[11] showing bites per person per night over a 2-year period. Lines show the fitted curves.**

Note that there were substantial differences between the two years in the measured bites per night by *An. arabiensis* and hence the fitted curve represents an average for these years.

### Matimbwa, Tanzania

Matimbwa is a village in the Bagamoyo area of coastal Tanzania. In this region there are typically two rainy seasons from March to July and from November to December. In the period studied by Shiff et al. [12] (1992), in Matwimba and other nearby villages *An. gambiae s.s.* was the major species during the long rainy season whilst *An. funestus* was dominant during the short rainy season later in the year. *An. arabiensis* is also present mainly in the latter half of the year, apparently favoring drier and hotter conditions [12].

The overall intensity of transmission in this village is high, with an annual measured EIR of 703 ibppy. Consequently parasite prevalences are also high, with 74.5% of children aged 6 to 40 months of age positive by microscopy during a cross-sectional survey undertaken in June 1992 [12].

**Figure S4.5 – Fit of seasonality curve to EIR measured in Matwimba, Tanzania. Dots: data adapted from Shiff et al. (1995) [12] showing measured monthly EIR over a 1-year transmission season. Lines show the fitted curves.**

### Kjenjojo Kasiina, Uganda

Malaria is endemic in the highlands of Kenya and Uganda transmission is well studied in these areas. Vector densities were collected in Kjenjojo, in southwestern Uganda from June 2001 to May 2002 [13]. In this hilly, rural area, the landscape is dominated by grassland and coffee and tea are grown. Temperatures ranged from 18 to 30 °C, and there was 918mm of rainfall in the year. The village is sited at 1,312 metres above sea level. The EIR ranged from 0.04 to 0.11 per person per night. *An gambiae s.s.* dominated transmission in this area, contributing 65.4% of the EIR, with *An. funestus* contributing the rest. Both species were present throughout the year, except at the end of the long dry season (February), when *An. funestus* was absent. There was no *An. arabiensis* in the area. *P. falciparum* prevalence was measured at 67.8% (58.0-77.6) in 1-9 year olds.

**Figure S4.6 - - Fit of seasonality curve to biting data from Kjenjojo Kasiina, Uganda. Dots: data adapted from Okello et al. (2006) [13] showing bites per person per night over a 1-year period. Lines show the fitted curves.**

## References

1. Binka FN, Morris SS, Ross DA, Arthur P, Aryeetey ME (1994) Patterns of malaria morbidity and mortality in children in northern Ghana. Trans R Soc Trop Med Hyg 88: 381-385.

2. Chandramohan D, Owusu-Agyei S, Carneiro I, Awine T, Amponsa-Achiano K, et al. (2005) Cluster randomised trial of intermittent preventive treatment for malaria in infants in area of high, seasonal transmission in Ghana. BMJ 331: 727-733.

3. Leimkugel J, Hodgson A, Forgor AA, Pfluger V, Dangy JP, et al. (2007) Clonal waves of Neisseria colonisation and disease in the African meningitis belt: eight- year longitudinal study in northern Ghana. PLoS Med 4: e101.

4. Koram KA, Owusu-Agyei S, Fryauff DJ, Anto F, Atuguba F, et al. (2003) Seasonal profiles of malaria infection, anaemia, and bednet use among age groups and communities in northern Ghana. Trop Med Int Health 8: 793-802.

5. Oduro AR, Koram KA, Rogers W, Atuguba F, Ansah P, et al. (2007) Severe falciparum malaria in young children of the Kassena-Nankana district of northern Ghana. Malar J 6: 96.

6. Appawu M, Owusu-Agyei S, Dadzie S, Asoala V, Anto F, et al. (2004) Malaria transmission dynamics at a site in northern Ghana proposed for testing malaria vaccines. Trop Med Int Health 9: 164-170.

7. Hay SI, Guerra CA, Gething PW, Patil AP, Tatem AJ, et al. (2009) A world malaria map: Plasmodium falciparum endemicity in 2007. PLoS Med 6: e1000048.

8. Cohuet A, Simard F, Wondji CS, Antonio-Nkondjio C, Awono-Ambene P, et al. (2004) High malaria transmission intensity due to Anopheles funestus (Diptera: Culicidae) in a village of savannah-forest transition area in Cameroon. J Med Entomol 41: 901-905.

9. Karch S, Garin B, Asidi N, Manzambi Z, Salaun JJ, et al. (1993) [Mosquito nets impregnated against malaria in Zaire]. Ann Soc Belg Med Trop 73: 37-53.

10. Hay SI, Sinka ME, Okara RM, Kabaria CW, Mbithi PM, et al. (2010) Developing global maps of the dominant *Anopheles* vectors of human malaria. PLoS Med: in press.

11. Mendis C, Jacobsen JL, Gamage-Mendis A, Bule E, Dgedge M, et al. (2000) Anopheles arabiensis and An. funestus are equally important vectors of malaria in Matola coastal suburb of Maputo, southern Mozambique. Med Vet Entomol 14: 171-180.

12. Shiff CJ, Minjas JN, Hall T, Hunt RH, Lyimo S, et al. (1995) Malaria infection potential of anopheline mosquitoes sampled by light trapping indoors in coastal Tanzanian villages. Med Vet Entomol 9: 256-262.

13. Okello PE, Van Bortel W, Byaruhanga AM, Correwyn A, Roelants P, et al. (2006) Variation in malaria transmission intensity in seven sites throughout Uganda. Am J Trop Med Hyg 75: 219-225.
