# Supplementary material for: Reducing Plasmodium falciparum Malaria Transmission in Africa: A Model-Based Evaluation of Intervention Strategies
Source: PLoS Med. 2010 Aug 10;7(8):e1000324. doi: 10.1371/journal.pmed.1000324 (PMC2919425; doi:10.1371/journal.pmed.1000324)
Supplement: Protocol S5 — Additional results and sensitivity analyses. (1.26 MB DOC) [file pmed.1000324.s009.doc]

**Reducing *Plasmodium falciparum* malaria transmission in Africa: a model-based evaluation of intervention strategies**

Jamie T Griffin^1^, T. Deirdre Hollingsworth^1^, Lucy C Okell^1^, Thomas S Churcher^1^, Michael White^1^, Wes Hinsley^1^, Teun Bousema^2^, Chris J Drakeley^2^, Neil M Ferguson^1^, María-Gloria Basáñez^1^, Azra C Ghani^1^.

1. *MRC Centre for Outbreak Analysis & Modelling, Department of Infectious Disease Epidemiology, Imperial College London*
2. *Department of Infectious Diseases, London School of Hygiene & Tropical Medicine*

# PROTOCOL S5

**ADDITIONAL RESULTS & SENSITIVITY ANALYSES**

## Additional Results

### Individual scenarios for LLIN use

Figure 3 in the main text showed summary outputs from the scenarios considering the impact of increasing coverage of LLINs alone in addition to the switch to use of ACT as first-line treatment from 2000 onwards. below shows the time series of parasite prevalence across all six transmission settings. Whilst an impact in terms of decreased prevalence is observed in all six settings, only in the lowest transmission setting is it possible to reduce parasite prevalence close to the 1% threshold with this intervention alone.

**Figure S5.1: Impact of LLINs alone in the six transmission settings**

### Sequencing of IRS and MSAT

We additionally considered the potential impact of limited rounds of IRS and MSAT either jointly or in sequence. Here we considered 3 annual rounds of each. In the low transmission setting of Kejenjo Kasiina, Uganda, limited rounds are sufficient to further speed declines in transmission that would occur with LLINs alone (Figure S5.2**a**). In the moderate and high transmission settings, if limited rounds are used, then whilst temporary declines in parasite prevalence are observed, because these are insufficient over 3 years to drive the effective reproduction number below 1, parasite prevalence rebounds to the levels that would occur without additional IRS or MSAT (Figure S5.2). In some cases a short rebound is observed which is due to the temporary loss of immunity associated with reducing transmission early on. In general, there is no clear difference between the ordering of IRS and MSAT in the scenarios considered here. However, further exploration of a wider range of scenarios is warranted.

**Figure S5.2: Impact of sequencing of 3 rounds of IRS and 3 rounds of MSAT on parasite prevalence in the 6 transmission settings**

### High LLIN coverage

In the main text we considered optimistic scenarios in which up to 80% coverage of LLINs could be achieved coupled with twice yearly IRS and MSAT. However, even with such high intensity intervention programs it was not possible to reduce parasite prevalence below the 1% threshold for our two high transmission settings, KND, Ghana and Matimbwa, Tanzania. Whilst higher coverage levels may be unrealistic, we undertook further runs to test whether at either 90% or 95% LLIN coverage and with perfect adherence, it would be possible to reduce transmission to low levels. IRS and MSAT coverage were also increased to 90 or 95% for these runs. In both settings a further decrease is observed but transmission is sustained (Figure S5.3).

**Figure S5.3: Increasing coverage levels under scenarios of perfect adherence to LLIN use, coupled with twice yearly IRS and MSAT in A) KND, Ghana and B) Matimbwa, Tanzania**

## Sensitivity Analysis

### Duration of anti-infection immunity

When estimating the transmission model parameters, the parameter *d_B_* which determines the time-scale over which immunity to infection is lost if transmission is interrupted was fixed at 10 years since the data we have are not informative about its value. The parameter which governs the loss of immunity to disease was also fixed, but its value is less important for the results presented in this paper as we are not considering morbidity when assessing the interventions. Immunity leading to faster clearance of parasites is assumed to be acquired purely with age, not with exposure, and so is not lost as transmission is reduced. We explored the impact that three different values of *d_B_* would have on the results: a shorter value of 5 years, plus longer values of 20 or 30 years. For each value, the transmission model was re-fitted to the data described in Protocol S3. The assumed time-scale over which immunity is lost has a substantial impact on the extent and speed of the rebound in prevalence that occurs after the initial drop (Figure S5.4), and so none of the results should be taken as predictions of the time course of prevalence during an elimination campaign.

**Figure S5.4: Changing the parameter *d*_B_ with A) LLINs only in Kinkole, DRC; B) LLINs with yearly IRS and MSAT in KND, Ghana**

### Exophilic behaviour of An. Arabiensis

In the scenarios presented in the main text, our results suggested that it would be difficult to reduce parasite prevalence below the 1% threshold in Maputo, Mozambique because of the presence of *An. arabiensis*. This was due to the relatively lower effect of IRS on *An.arabiensis* that results from the low degree of endophilic behaviour (16%) assumed for this vector. However, as estimates of this parameter are from a limited number of studies, here we explore the impact of 3 alternative values – 49% as measured in [1], an intermediate value of 25% and for comparison with the other species a value of 86%. As expected there is a clear increasing efficacy of IRS-based intervention programs as the degree of endophilic behaviour assumed for *An.arabiensis* is increased (Figure S5.5). However, at the other measured value for this parameter (49% versus the 16% assumed in the main text), there is no change to our conclusion that additional tools are likely to be necessary in this setting.

**Figure S5.5: Predicted impact of IRS-based intervention programs in Maputo, Mozambique for different assumptions regarding the degree of endophilic behaviour of *An.arabiensis.***

### Killing and repellency effect of LLINs

The impact of LLIN distribution on transmission is dependent on the assumptions for the degree of killing and repellency effect assumed in our model. Here we considered an alternative set of parameters in which the LLINs have a higher degree of repellency and an associated lower killing effect. These are the set of parameters in brackets summarised in Table S5.1 below extracted from Table S3.4 in Protocol S3.

**Table S5.1**: Parameters for LLIN effect. Alternative parameters examined here are shown in brackets.

| **Definition** | **Model**  **parameter** | **Best estimate with alternative estimates where available in brackets** | | | **References** |
| --- | --- | --- | --- | --- | --- |
|  |  | ***An. funestus*** | ***An. arabiensis*** | ***An. gambiae s.s.*** |  |
| Cycle repeating probability for LLINs | $r_{N0}$ | 0.56 (0.69) | 0.48 (0.23) | 0.56 (0.52) | [2-4] |
| Successful feeding with LLINs | $s_{N0}$ | 0.03 (0.14) | 0.39 (0.33) | 0.03 (0.16) | [2-4] |
| Insecticide mortality probability for LLINs | $d_{N0}$ | 0.41 (0.17) | 0.13 (0.44) | 0.41 (0.32) | [2-4] |
| Baseline repeating action for LLINs | $r_{NM}$ | 0.24 | 0.10 | 0.24 | [2, 3] |
| Half-life of LLIN efficacy | Log(2)/$\gamma_{N}$ | 2.64 years | | | [5] |

In the low transmission setting of Kjenjojo, Uganda, under our baseline assumptions it was possible to reduce transmission to very low levels if a program of LLIN distribution with high levels of adherence was attained. However, with an alternative insecticide-treated net with lower killing effect, transmission is sustained (Figure S5.6**A**). In this setting, the effective reproduction number is close to 1 following the scale-up of LLINs and hence small variations in the killing effect of LLINs can make a difference. In contrast, in the moderate transmission setting of Kinkole, DRC, LLINs alone or in combination with yearly IRS and MSAT are insufficient to bring the effective reproduction number close to 1. Thus the impact of the parameters determining LLIN efficacy is lower (Figure S5.6**B**).

**Figure S5.6: Impact of LLIN distribution in two transmission settings under alternative assumptions for the killing and repellency effect of the nets.**

### Alternative insecticides for IRS

In the main text we used characteristics for the IRS interventions based on the use of DDT as currently recommended by WHO. Here we also consider the impact of an alternative insecticide, namely lambdacyhalothrin. The comparative parameters for DDT and lambdacyhalotrhin are shown in Table 5.2 below extracted from Table 3.4 in Protocol S3.

**Table S5.2** Parameters for different IRS insecticides.

| **Definition** | **Model**  **parameter** | **Best estimate with alternative estimates where available in brackets** | | | | **Refs** |
| --- | --- | --- | --- | --- | --- | --- |
|  |  | ***An. funestus*** | ***An. arabiensis*** | ***An. gambiae s.s.*** | |  |
| Cycle repeating probability for IRS with DDT | $r_{S0}$ | 0.63 | 0.60 | | 0.60 | [6] |
| Cycle repeating probability for lambdacyhalothrin | $r_{S0}$ | 0.207 | | | | [7] |
| Half-life of IRS DDT efficacy | Log(2)/$\gamma_{S}$ | 0.5 years | | | | [8] |
| Half-life of IRS lambdacyhalothrin efficacy | Log(2)/$\gamma_{S}$ | 0.13 years | | | | [7] |

The impact of each insecticide as a single IRS program undertaken yearly in addition to LLIN distribution is shown in Figure S5.7. Whilst DDT has a lower estimated killing effect compared to lambdacyhalothrin, its slower estimated decay rate makes it more effective as an insecticide in programs in which spraying occurs yearly.

**Figure S5.7: Effect of alternative insecticides on the impact of an IRS program in two transmission settings.**

### High intensity MSAT

In some transmission settings (for example recent interventions in Vanuatu) more frequent MDA or MSAT rounds have been undertaken. We considered this as an additional more intensive intervention that could be undertaken in the high transmission settings of KND, Ghana and Matimbwa, Tanzania in which twice yearly IRS and MSAT coupled with LLIN distribution was unable to reduce prevalence to below 1%. In both settings, monthly MSAT at 80% coverage coupled with twice yearly IRS and LLIN, both with 80% coverage, it is possible to reduce transmission to very low levels provided a random sample of individuals is screened and treated each month (**Figure S5.8**). However, even with such an intense program there is still a continual low prevalence of parasites in the populations and thus it is likely that this program would need to be continued for many years. Notably, if 80% coverage is achieved but instead of random distribution the same people access the MSAT programs, prevalence is not reduced by the same degree.

**Figure S5.8: Impact of frequent MSAT on transmission in two settings.**

### Pre-erythrocytic Vaccine Characteristics

The results in the main text are based on characteristics of the RTS’S/AS01 vaccine currently in Phase III trials. This is the only vaccine that is likely to become licensed in the near future. However, we also considered a theoretical vaccine with higher efficacy and a longer half-life. In general, increasing both efficacy and the half-life increases the effect of a vaccination campaign in the expected manner. Here we show the impact that a 95% efficacious PE vaccine (for example an optimistic efficacy for the Sanaria vaccine [9]) coupled with a 10 year half-life could have. Our scenarios for mass vaccination further assume that repeated vaccination occurs every 3 years thereby enabling high levels of protection in the vaccinated community. For all scenarios 90% coverage of the vaccination campaign is assumed.

With such a theoretical vaccine, mass vaccination alone could eliminate infection in our low transmission setting of Kjenjojo, Uganda **(Figure S5.9A**). In all three moderate transmission settings yearly rounds of IRS and MSAT and a mass vaccination campaign coupled with distribution of LLINs could reduce transmission to low levels (**Figure S5.9 B-D**). In both high transmission settings, such a program with twice yearly MSAT and IRS could also achieve rapid reductions, although in these settings transmission is likely to continue and hence the interventions would need to be sustained (**Figure S5.9E-F**).

**Figure S5.9 Impact of a theoretical high efficacy long duration pre-erythrocytic vaccine.**

## References

1. Molineaux, L., G.R. Shidrawi, J.L. Clarke, J.R. Boulzaguet, and T.S. Ashkar, *Assessment of insecticidal impact on the malaria mosquito's vectorial capacity, from data on the man-biting rate and age-composition.* Bull World Health Organ, 1979. **57**(2): p. 265-74.

2. Curtis, C.F., J. Myamba, and T.J. Wilkes, *Comparison of different insecticides and fabrics for anti-mosquito bednets and curtains.* Med Vet Entomol, 1996. **10**(1): p. 1-11.

3. Lines, J.D., J. Myamba, and C.F. Curtis, *Experimental hut trials of permethrin-impregnated mosquito nets and eave curtains against malaria vectors in Tanzania.* Med Vet Entomol, 1987. **1**(1): p. 37-51.

4. Mathenge, E.M., J.E. Gimnig, M. Kolczak, M. Ombok, L.W. Irungu, and W.A. Hawley, *Effect of permethrin-impregnated nets on exiting behavior, blood feeding success, and time of feeding of malaria mosquitoes (Diptera: Culicidae) in western Kenya.* J Med Entomol, 2001. **38**(4): p. 531-6.

5. Mahama, T., E.J. Desiree, C. Pierre, and C. Fabrice, *Effectiveness of permanet in Cote d'Ivoire rural areas and residual activity on a knockdown-resistant strain of Anopheles gambiae.* J Med Entomol, 2007. **44**(3): p. 498-502.

6. Smith, A. and D.J. Webley, *A verandah-trap hut for studying the house-frequenting habits of mosquitoes and for assessing insecticides. 3. The effect of DDT on behavior and mortality.* Bull Entomol Res, 1969. **59**(1): p. 33-46.

7. N'Guessan, R., V. Corbel, M. Akogbeto, and M. Rowland, *Reduced efficacy of insecticide-treated nets and indoor residual spraying for malaria control in pyrethroid resistance area, Benin.* Emerg Infect Dis, 2007. **13**(2): p. 199-206.

8. World Health Organisation, *Pesticides and their application*. 2006, World Health Organisation, : Geneva.

9. Hoffman, S.L., P.F. Billingsley, E. James, A. Richman, M. Loyevsky, T. Li, et al., *Development of a metabolically active, non-replicating sporozoite vaccine to prevent Plasmodium falciparum malaria.* Human vaccines. **6**(1).
