# Supplementary material for: Reducing Plasmodium falciparum Malaria Transmission in Africa: A Model-Based Evaluation of Intervention Strategies
Source: PLoS Med. 2010 Aug 10;7(8):e1000324. doi: 10.1371/journal.pmed.1000324 (PMC2919425; doi:10.1371/journal.pmed.1000324)
Supplement: Protocol S6 — User-friendly software for model runs. (0.17 MB DOC) [file pmed.1000324.s010.doc]

**Reducing *Plasmodium falciparum* malaria transmission in Africa: a model-based evaluation of intervention strategies**

Jamie T Griffin^1^, T. Deirdre Hollingsworth^1^, Lucy C Okell^1^, Thomas S Churcher^1^, Michael White^1^, Wes Hinsley^1^, Teun Bousema^2^, Chris J Drakeley^2^, Neil M Ferguson^1^, María-Gloria Basáñez^1^, Azra C Ghani^1^.

1. *MRC Centre for Outbreak Analysis & Modelling, Department of Infectious Disease Epidemiology, Imperial College London*
2. *Department of Infectious Diseases, London School of Hygiene & Tropical Medicine*

# PROTOCOL S6

**USER-FRIENDLY SOFTWARE FOR MODEL RUNS**

The user-friendly software that is provided free from our website ([www.imperial.ac.uk/medicine/malariatools](http://www.imperial.ac.uk/medicine/malariatools)) enables readers to reproduce the results presented in the paper, as well as to explore other combinations of interventions. This software is provided as a Beta version and updates/fixes will appear periodically on the website.

In this initial release, the model can only generate runs for the six transmission settings described in the main text. Future releases will allow users to input data from their own settings.

## Installation

Full installation details are provided on the Malaria Tools website. Once installed, the application will appear in the Windows menu bar. On running the program a single user screen will appear as shown below.

*
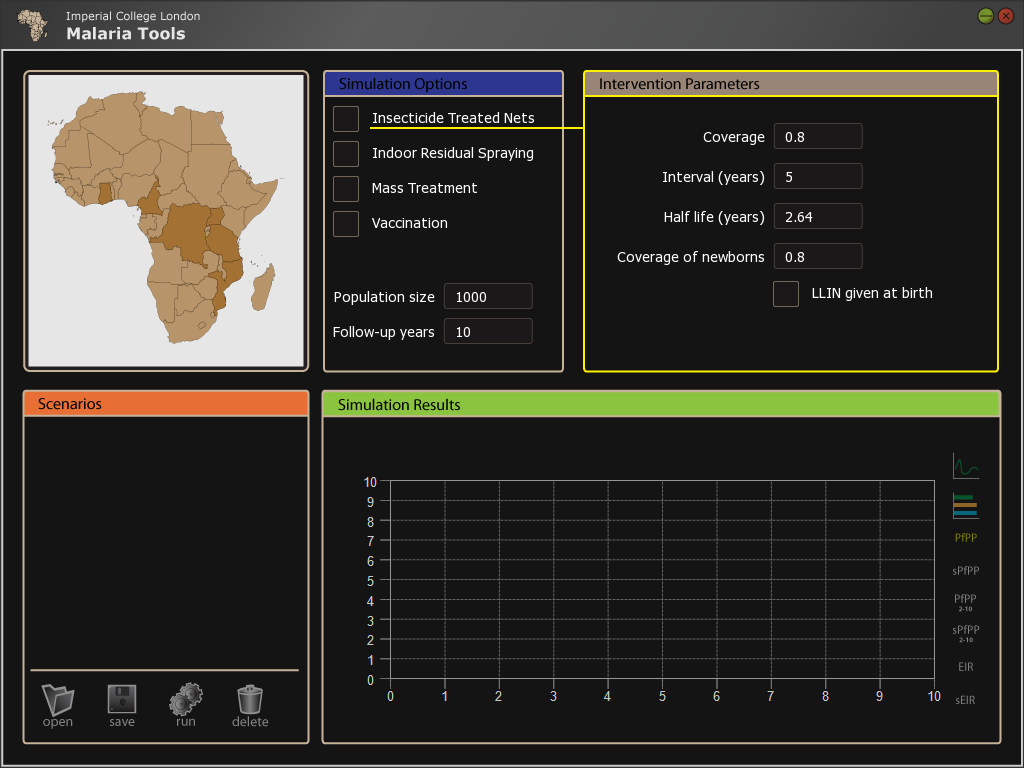
*

**NOTE**: In this initial release the application screen has a fixed size of 1024x768 pixels. This cannot be changed at the moment.

## Changing the transmission setting

The left-hand map allows the user to select one of the six pre-defined transmission settings that correspond to those in Table 1 of the main paper. By scrolling over the map the countries are highlighted and a red dot indicates the specific location of the transmission setting. By a single click the highlighted setting is selected and the name appears in the bottom left-hand corner of the map. This setting is used for all subsequent runs until changed.

## Simulation Characteristics

Two options are available for the baseline simulation characteristics.

**Population size** – the size of population simulated. The default value is 1000 which enables reasonably accurate runs to be simulated in at most a few minutes. The results in the main text are based on a population of 10,000 which is sufficiently large to obtain robust results but will take substantially longer to perform. Larger population sizes are not recommended. Runtime is slower for settings with higher transmission intensity (e.g. Matimbwa, Tanzania or KND, Ghana). In all cases, the output produced is the mean of 10 stochastic simulations each with the specified population size.

**Follow-up years** – the time over which the scenarios are performed starting from 2010 (year 0). Runs with longer follow-up will be slower to perform.

## Choosing interventions and their parameters

The “Simulation Options” box shows the list of currently implementable interventions; by clicking on the boxes to the left of each intervention these can be switched on and off. By clicking on the intervention name the “Intervention Parameters” box for that intervention is shown. Each of the parameters in this box can be varied by the user.

If an intervention parameter value is entered outside the bounds allowable by the program the value is clipped to the exceeded boundary. For example, if a coverage level >1 the value with will be clipped to 1 and if a value <0 is entered the value will be clipped to 0.

A summary of the parameters that can be varied for each intervention is given below.

### Insecticide Treated Nets (LLIN)

All scenarios include a scale-up in LLINs from 0% to 20% coverage between 2000 and 2010.

**Coverage** – indicates the proportion of individuals in the population receiving nets and should be between 0 and 1. All runs assume random distribution. The scenarios are started in 2010 when we assume 20% coverage has already been achieved. Thus setting the coverage level below 20% will result in an increase in transmission.

**Interval (years)** – indicates the interval between distribution and re-distribution of nets. In the results in the main text nets are distributed every 5 years.

**Half-life (years)** – indicates the half-life for insecticide efficacy. The default value is 2.64 years based on data for permanets.

**LLIN at birth / Coverage of newborns –** If LLIN at birth is selected nets are additionally distributed at birth to the proportion of newborns entered here (between 0 and 1)

### Indoor Residual Spraying

**Coverage** – indicates the proportion of individuals in the population sleeping in houses in which IRS is implemented and should be between 0 and 1. All runs assume random distribution

**Interval (years)** – indicates the interval between spraying and re-spraying. For example, a value of 1 indicates spraying every year, a value of 0.5 every 6 months and a value of 2 every other year. The default value is every year. The seasonal timing of yearly IRS is set at the time determined to be optimal. More frequent spraying is distributed evenly throughout the year starting at the optimal time. Less frequent spraying is started at the optimal time in the first year and subsequently scheduled according to the user frequency entered.

**Half-life (years)** – indicates the half-life for insecticide efficacy. The default value is 6 months (0.5 years) based on data for DDT.

**Start year** – indicates the year IRS spraying is introduced. Year zero corresponds to 2010. All scenarios include a scale-up in LLINs from 0% to 20% coverage between 2000 and 2010.

### Mass Treatment

**Coverage** – indicates the proportion of individuals in the population who receive the intervention. For MDA this is the proportion receiving treatment, whilst for MSAT it is the proportion screened, following which all who are parasite positive using microscopy are treated.

**Interval (years)** – indicates the interval between repeated rounds. For example, a value of 1 indicates mass treatment every year, a value of 0.5 every 6 months and a value of 2 every other year. The default value is every year. The seasonal timing of yearly MDA/MSAT is set at the time determined to be optimal. More frequent rounds are distributed evenly throughout the year starting at the optimal time. Less frequent rounds are started at the optimal time in the first year and subsequently scheduled according to the user frequency entered.

**Start year** – indicates the year MDA/MSAT is introduced. Year zero corresponds to 2010. All scenarios include a scale-up in LLINs from 0% to 20% coverage between 2000 and 2010.

**MDA/MSAT** – 0 indicates MDA and 1 indicates MSAT

**MDA Drug** – 0 indicates a non-ACT (sulfadoxine pyrimethamine) and 1 an ACT (artemether-lumefantrine)

Within this section we have also included IPTi distributed through the Expanded Program on Immunization (EPI) although this is not included in the main text as it has no noticeable impact on transmission. When selected options for this are:

**Coverage via EPI** – the proportion of infants receiving IPTi at the EPI ages (3, 4 and 5 months)

**IPTi Drug** - 0 indicates a non-ACT (sulfadoxine pyrimethamine) and 1 an ACT (artemether-lumefantrine)

### Pre-erythrocytic vaccine (PEV)

**Mass PEV** – Indicates that the vaccine is distributed uniformly across the population

**PEV start** – indicates the year PEV is introduced. Year zero corresponds to 2010. All scenarios include a scale-up in LLINs from 0% to 20% coverage between 2000 and 2010.

**PEV coverage** - indicates the proportion of individuals in the population who receive the vaccine via mass administration, between 0 and 1.

**PEV interval** - indicates the interval in years between vaccination and boosters for mass vaccination. The default value is every 3 years. This is based on a vaccine with a half-life of 3 years.

**EPI PEV** – Indicates that the vaccine is distributed to infants via EPI either with or without additional mass vaccination

**PEV at EPI coverage** - indicates the proportion of infants in the population who receive the vaccine via EPI, between 0 and 1.

## Running the model

Click on the Run icon in the bottom left-hand corner to run the selected simulation. While the model runs the screen will fade and a progress monitor will appear.

## Plotted Output

Six output statistics can be plotted on the screen:

**PfPP** – *Plasmodium falciparum* parasite prevalence across all age-groups

**sPfPP** – *Plasmodium falciparum* parasite prevalence across all age-groups smoothed to remove seasonal fluctuations

**PfPP2-10** – *Plasmodium falciparum* parasite prevalence in children aged 2 to 10 years

**sPfPP2-10** – *Plasmodium falciparum* parasite prevalence in children aged 2 to 10 years smoothed to remove seasonal fluctuations

**EIR** – annual entomological inoculation rate

**sEIR** – annual entomological inoculation rate smoothed to remove seasonal fluctuations

These different outputs can be displayed by clicking on the appropriate icon to the right of the Simulation Results box.

The top two icons allow the user to switch between the types of graph displayed:

**Line graph** – displays the statistic over time from the start to end of the simulation

**Bar chart** – displays the statistic at the end of the run as a bar chart

## Importing, saving and deleting runs

The scenarios box lists the runs that are currently loaded. By clicking on these the output from the runs will be displayed in the Simulation Results. Runs can be deleted using the delete button.

Model runs can be saved to text files by first selecting the model runs to be saved and then using the save button. The location of the saved files is input by the user. Three output files are saved for each selected scenario once the user enters a file name:

[Selected Name].[Scenario Name].txt

[Selected Name].[Scenario Name].param.txt

[Selected Name].[Scenario Name].output.txt

To import previously saved model runs the user should use the load button and select the file type 1 from the above to import the run parameters and output.

## Saved Output

[Selected Name].[Scenario Name].param.txt contains the scenario parameters that have been selected.

[Selected Name].[Scenario Name].output.txt contains the following output in columns:

| **Column heading** | **Description** |
| --- | --- |
| year | Year of simulation relative to 2010. Output starts from -2 (2008). |
| EIRY_mean | Annual EIR in adults |
| slide_pos_mean | Mean parasite prevalence by microscopy across the whole population |
| slide_pos_2_10_mean | Mean parasite prevalence by microscopy in 2 to 10 year-olds |
| slide_pos_a_mean, a=1 to 6 | Mean parasite prevalence by microscopy in 6 age-groups:  1 = [0-1); 2=[1,2); 3=[2-5), 4=[5-10), 5=[10-15), 6=15+ |
| total_pos_mean | Mean parasite prevalence by PCR across the whole population |
| total_pos_2_10_mean | Mean parasite prevalence by PCR in 2 to 10 year-olds |
| total_pos_a_mean, a=1..6 | Mean parasite prevalence by PCR in 6 age-groups:  1 = [0-1); 2=[1,2); 3=[2-5), 4=[5-10), 5=[10-15), 6=15+ |
| EIRY_mean_smooth | Smoothed annual EIR in adults |
| slide_pos_mean_smooth | Smoothed mean parasite prevalence by microscopy across the whole population |
| slide_pos_2_10_mean_smooth | Smoothed mean parasite prevalence by microscopy in 2 to 10 year-olds |
| slide_pos_a_mean_smooth, a=1..6 | Smoothed mean parasite prevalence by microscopy in 6 age-groups:  1 = [0-1); 2=[1,2); 3=[2-5), 4=[5-10), 5=[10-15), 6=15+ |
| total_pos_mean_smooth | Smoothed mean parasite prevalence by PCR across the whole population |
| total_pos_2_10_mean_smooth | Smoothed mean parasite prevalence by PCR in 2 to 10 year-olds |
| total_pos_a_mean_smooth, a=1..6 | Smoothed mean parasite prevalence by PCR in 6 age-groups:  1 = [0-1); 2=[1,2); 3=[2-5), 4=[5-10), 5=[10-15), 6=15+ |

Smoothed output is the mean over the previous year. Therefore in the first year this does not appear and the missing data is represented by -999 in the text file.

## Interrupting the program

The program can be interrupted while running a simulation by using the ESC key.
